# Black Rice Performance Under Water Deficit Conditions and Genotype X Environment Interactions

**DOI:** 10.3390/plants14223459

**Published:** 2025-11-12

**Authors:** Aloysha Brunet-Loredo, Abdelhalim Elazab, Karla Cordero-Lara, Paula Careaga, Miguel Garriga

**Affiliations:** 1 Department of Plant Production, Faculty of Agronomy, University of Concepcion, Avenida Vicente Mendez 595, Chillán 3812120, Chile; abrunet@udec.cl (A.B.-L.); akhaled@udec.cl (A.E.); agro.paulacr@gmail.com (P.C.); 2 Institute of Agricultural Research, Regional Research Center Quilamapu, Avenida Vicente Mendez 515, Chillán 3812120, Chile

**Keywords:** grain yield, grain quality, non-flooding irrigation, heritability

## Abstract

Rice is a staple food grown worldwide. While white rice varieties have been extensively studied, there is limited information on the performance of pigmented rice genotypes and their tolerance to water deficit. This study evaluated nineteen black rice genotypes and one white cultivar over three years under contrasting water regimes: traditional flooding and non-flood irrigation (NFI). Genotype–environment interactions and their impact on agronomic, yield, and grain quality traits were assessed. Black genotypes under NFI showed reduced flowering and grain quality. The average yield was 31% lower than the white cultivar. Significant genetic correlations were found between grain yield and days to anthesis (DSA), grain weight (TGW), chalkiness (CHA), and translucency (TRAN), with high broad-sense heritability (H^2^ > 0.9). Most traits exhibited high heritability (H^2^ > 0.7), indicating strong genetic stability. Grain yield (GR) was highly and negatively correlated with percent sterility (PS) (r = −0.84) and had a heritability of 0.76. Environmental conditions significantly influenced yield, confirming the potential for selecting water stress–tolerant genotypes. These findings provide valuable insights into black rice breeding and optimizing water management practices to support sustainable production. To our knowledge, this study is the first to evaluate the performance of a diverse set of black rice genotypes across multiple seasons under contrasting water regimes in a Mediterranean environment.

## 1. Introduction

Rice (*Oryza sativa* L.) is one of the most important staple foods worldwide, providing nearly 25% of the daily caloric and protein intake for the global population [1,2,3]. In 2023, rice production reached approximately 800 million tons across 143 countries, with a cultivated area of 168 million hectares [1]. Among its numerous varieties, black rice has attracted growing attention due to its distinctive dark pigmentation and high concentration of bioactive compounds. The pericarp of black rice contains substantial amounts of phenolic compounds, particularly anthocyanins, responsible for its deep purple coloration and potent antioxidant activity [4,5,6]. In addition, black rice is rich in flavanones, phenolic acids, vitamins, fatty acids, and carotenoids [5,6,7], which confer recognized health promoting properties, supporting its classification as a functional food [8]. For centuries, pigmented rice varieties have been used for medicinal purposes in countries such as China and India for their nutritional and medicinal benefits [6,7]. In addition, black rice is described as a versatile ingredient, as it can be transformed into various products, including bread, cookies, noodles, and beverages, which is an attractive feature for consumers [6,8]. Moreover, extracts rich in anthocyanins from black rice serve as a natural food pigment [6]. In fact, pure black rice powder is utilized as a colorant (E163) and in dietary supplements, a market that is experiencing rapid expansion [9].

Despite its nutritional relevance, rice production faces severe sustainability challenges, mainly related to increasing water scarcity. Water stress is the main limiting factor for plant growth, influencing physiological, biochemical, and molecular processes, and ultimately affecting yield [10,11,12,13]. This stress is particularly detrimental for rice, which is typically cultivated under flooded conditions [14]. While different management practices and water conservation techniques can sustain high yields in many rice growing regions [15], they are typically implemented in areas where cold temperatures are not a limiting factor. Water stress in rice leads to wilting, increased senescence, stunted growth, leaf rolling, and diminished photosynthesis, ultimately resulting in lower grain yields [12,16,17]. Furthermore, drought conditions significantly affect several grain quality traits, such as milling rate [18], protein and amylose content [19], antioxidant capacity [20,21,22], mineral nutrient levels [23,24,25], and cooking quality [18].

Efficient water management is therefore crucial for sustainable rice cultivation [26,27,28]. In Chile, rice is traditionally grown under flooded conditions, requiring over 18,000 m^3^ of water per hectare [14]. However, local rice production faces significant challenges in crop sustainability, including increasing water scarcity, primarily driven by climate change [29]. These constraints highlight the need to develop cultivars that combine high yield with improved water use efficiency, as well as to adopt innovative irrigation strategies such as non-flooding irrigation (NFI), could play a key role in conserving water resources [14], contributing to maintaining crop productivity and sustainability.

Under current climate and water scarcity scenario, developing black rice cultivars adapted to such conditions is essential to ensure resilient and sustainable crop production. Achieving this goal requires exploiting the existing genetic variability as a tool for adaptation to changing environments [30,31]. Evaluating different genotypes under both optimal and water-scarce conditions is crucial to assess yield and understand how the environment affects productivity and grain quality. Many productive and quality traits exhibit continuous or quantitative phenotypic variation and are typically controlled by multiple genes with diverse genetic effects and/or influenced by the environment [31], which makes the development of high-yielding drought-adapted rice genotypes a complex task [13,32].

Specific traits in rice have been identified as influential in maintaining yield stability under contrasting environments. According to Qiu et al. [33], plant height has been reported to correlate positively with grain productivity. Additionally, grain discoloration negatively affects grain morphology, decreasing grain weight and overall yield, thus impacting yield stability [34]. Studies in cereals, including rice, have shown that grain yield is positively associated with biomass, harvest index, and grains per square meter in drought-resistant varieties [35]. Furthermore, yield loss and reduction in harvest index under drought stress have been associated with decreased fertile panicle rate, biomass, and grain weight [36]. These findings highlight the significance of breeding programs in enhancing grain yield through the integration of traits such as plant height, thereby ensuring yield stability under variable environmental conditions [31,37].

Statistical tools are essential in breeding programs to identify genotypes with stable performance across environments. Multi-environment trials allow for assessment of genotype x environment interaction and estimation of heritability, providing insight into trait mechanisms. Moreover, they offer insights into the repeatability of genotypic responses by estimating broad-sense heritability [38]. High heritability (H^2^) is a key parameter in selection programs, as traits with high H^2^ can be effectively improved [39]. Previous studies in rice have shown that traits related to grain weight and quality often exhibit high heritability, making them ideal targets for genetic improvement strategies [33,34,37,40]. Moreover, traits with strong genetic correlations can enable indirect selection for desirable agronomic performance [41]. Despite significant advances in understanding drought adaptation in white rice, little is known about how black rice genotypes adjust their agronomic and quality-related traits under water-saving irrigation systems in temperate environments. A deeper understanding of how complex trait correlations respond to environmental variation is crucial to guide breeding programs focused on developing resilient black rice cultivars capable of maintaining yield and grain quality under non-flooded irrigation (NFI) conditions. In this context, a three-year field study was conducted to assess the performance of nineteen black rice genotypes under two contrasting water management systems (conventional flooding and NFI) in a Mediterranean zone of Chile. The analysis included one white rice cultivar to provide a comparative reference. The evaluation focused on agronomic, productive, and grain quality traits, with an emphasis on genotype x environment interactions.

## 2. Results

### 2.1. Effect of Genotype and Environment on Agronomic, Productive, and Quality Traits

The analysis of agronomic, productive, and quality traits in relation to the environment (water regime × year) revealed statistical differences (*p* < 0.05) among genotypes for DSA, GY, FGP, TGP, ST, PL, WG, TGWPa, TGWPo, CHA, and TRAN. The cultivar Zafiro-INIA exhibited the highest mean values for most traits, including DSA, GY, FGP, TGWPa, TGWPo, CHA, and TRAN. The interaction between genotype and environmental factors was also statistically significant for ST, PL, WG, TGWPa, TGWPo, and CHA (Table 1 and Table 2).

Regarding flowering time, significant differences were observed between Zafiro and Quila 297901, with a delay of 19 days in the former genotype. Additionally, the genotypes Quila 297901 and Quila 299801 flowered at 110 and 116 DSA, respectively, significantly earlier than Zafiro, which flowered at 129 DSA. Interestingly, despite their earlier flowering, pigmented rice genotypes achieved an average yield of 4.17 tons per hectare, which was 31% lower than the grain yield of Zafiro. The reduced yield of black genotypes in this study was largely attributed to a high percentage of floral sterility, which averaged 41.9%, compared to 30.3% in Zafiro (Table 1). Quila 292001 and FLQuila 93 showed sterility percentages of 35.4% and 37.7%, respectively, indicating some variability within the pigmented genotypes. In addition, the thousand-grain weight of pigmented genotypes was, on average, 18% lower than that of Zafiro, both for paddy rice and polished grains (Table 2), which also contributes to the lower yield of black rice genotypes compared to white rice. In terms of grain quality in pigmented genotypes, WG decreased by 2.2%, while ADD increased by 1.6%, compared to Zafiro. However, as expected, there were notable differences in grain appearance; CHA and TRAN decreased by 34.3% and 52%, respectively, in pigmented genotypes (Table 2).

The application of NFI resulted in significant changes in rice growth and productivity, affecting all the evaluated traits except PH and ADD (Table 1 and Table 2). On average, grain yields were 6.04 t ha^−1^ under flooding and 2.5 t ha^−1^ under NFI, representing a 2.4-fold decrease. This reduction in GY under NFI was accompanied by an 18.2% increase in DSA, ranging from 111.7 to 132.1 days under flooding and NFI, respectively. The decrease in GY under NFI was directly linked to reductions in FGP, TGP, TGWPa, and TGWPo, which decreased by 24.3%, 12.7%, 8.4%, and 8.1%, respectively. In addition, the reduction in yield and its components under NFI was accompanied by a 23.3% increase in ST. On the other hand, NFI increased WG, CHA, and TRAN by 14.8%, 2.3%, and 4.5%, respectively.

During the 2022 season, there was a notable decrease in yield under flooded conditions, reaching only half of that obtained in the 2021 and 2023 seasons. Nevertheless, the yields in 2022 were still higher than the average observed under NFI conditions. PL increased by 16.1% in the same season, TGP rose by 15.4%, and FGP decreased significantly by 32.5%. Additionally, ST was twice as high as the average observed in the other two seasons under flooded conditions. Grain weight showed a slight variation, with TGWPa and TGWPo decreasing by 0.8% and 1.34%, respectively, compared to the average of the other two years (Tables S1 and S2).

### 2.2. Correlation Analysis

**Figure 1:**
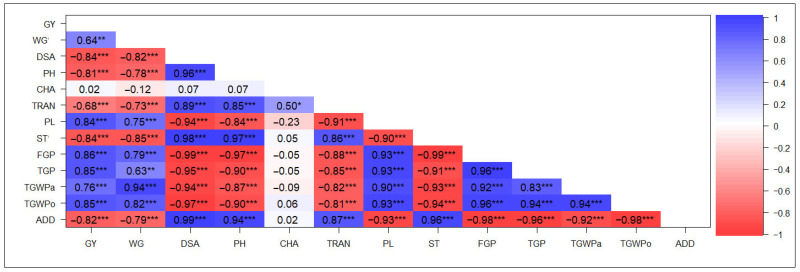
Pearson correlation analysis of agronomic, productive, and quality traits evaluated in 19 black rice genotypes and the white cultivar Zafiro grown under conventional flooding conditions over three consecutive years. * *p* ≤ 0.05, ** *p* ≤ 0.01, and *** *p* ≤ 0.001. GY: grain yield, WG: whole-grain yield percentage; DSA: days from sowing to anthesis; PH: plant height; CHA: grain chalkiness, TRAN: grain translucency; PL: panicle length; ST: sterility percentage; FGP: filled grain per panicle; TGP: total grain per panicle; TGWPa: thousand-grain weight of paddy rice; TGWPo: thousand-grain weight of polished rice; ADD: average degree of dispersion. *n* = 60.

Correlation analyses were performed between the agronomic, productive, and quality traits evaluated under water management conditions (flooding and NFI). Under flooded conditions, GY showed a high correlation with most evaluated traits, except for some quality traits. High positive correlations were observed between GY and TGP, FGP, PL, and TGWPo (r ≥ 0.84), while high negative correlations were found between GY and DSA, PH, ST, and ADD (r ≤ −0.81) (Figure 1). In addition, DSA showed high positive correlations with ST, PH, ADD, and TRAN (r ≥ 0.89), as well as high negative correlations with yield and its components (TGP, FGP, TGWPa), PL, and WG (r ≤ −0.82) (Figure 1).

**Figure 2:**
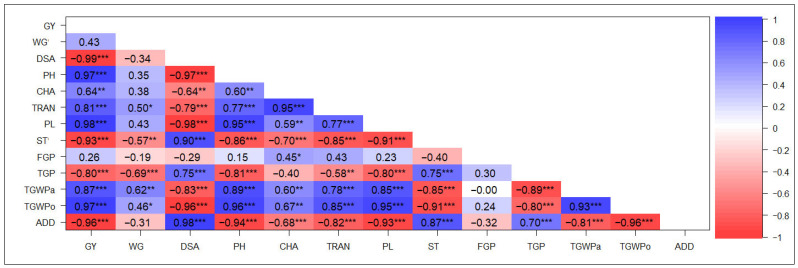
Pearson correlation analysis of agronomic, productive, and quality traits evaluated in 19 black rice genotypes and the white cultivar Zafiro grown under non-flooding irrigation conditions over three consecutive years. * *p* ≤ 0.05, ** *p* ≤ 0.01, and *** *p* ≤ 0.001. GY: grain yield, WG: whole-grain yield percentage; DSA: days from sowing to anthesis; PH: plant height; CHA: grain chalkiness; TRAN: grain translucency; PL: panicle length; ST: sterility percentage; FGP: filled grain per panicle; TGP: total grain per panicle; TGWPa: thousand-grain weight of paddy rice; TGWPo: thousand-grain weight of polished rice; ADD: average degree of dispersion. *n* = 60.

Under NFI conditions, GY showed a high correlation with most of the trials evaluated (Figure 2). As with flooding, high positive correlations were found between yield per hectare and thousand-grain weight (TGWPa and TGWPo) and PL (r ≥ 0.87), while high negative correlations were observed with DSA, ST, and ADD (r ≤ −0.93). However, unlike flood conditions, yield also showed high positive correlation with PH and TRAN (r ≥ 0.81), a high negative correlation with TGP, and a weak association with FGP. In addition, DSA showed a strong positive correlation with ST and ADD (r ≥ 0.9), as well as high negative correlations with PL and thousand-grain weight (r ≤ −0.83); however, unlike flooding, DSA showed a high positive correlation with TGP (r = 0.75) and high negative correlations with PH and TRAN (r ≤ −0.79). Under NFI conditions, ST was strongly correlated with GY, demonstrating more pronounced association compared to flooded conditions. Furthermore, ST showed a high positive correlation with TGP (r = 0.75) and strong negative correlations with PH and TRAN (r ≤ −0.85). The association of ST with FGP, while still negative, was weak under NFI (Figure 2).

### 2.3. Principal Component Analysis (PCA)

**Figure 3:**
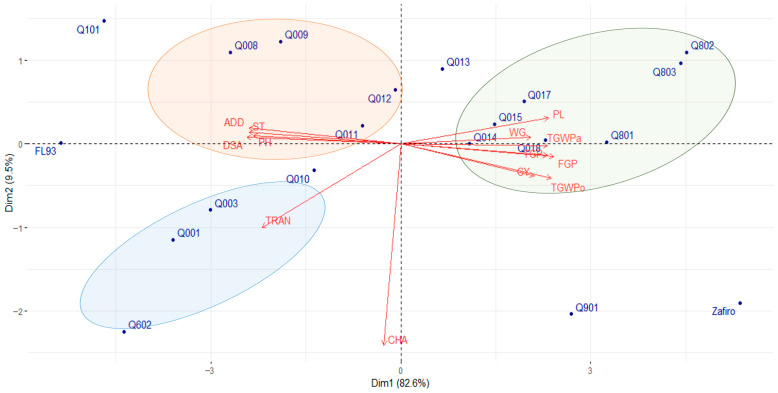
Principal components analysis (PCA) biplot of agronomic, productive, and quality traits of nineteen black rice genotypes and a white rice cultivar assessed under conventional flooding conditions over three consecutive years. GY: grain yield; PH: plant height; DSA: days from sowing to anthesis; WG: whole grain percentage; CHA: grain chalkiness; TRA: grain translucency; PL: panicle length; TGP: total grain per panicle; FGP: filled grain per panicle; ST: sterility percentage; TGWPa: thousand-grain weight of paddy rice; TGWPo: thousand-grain weight of polished rice; ADD: average degree of dispersion. Black rice genotypes; FL93: FQuila; 93; Q602: Quila 291602; Q001: Quila 292001; Q003: Quila 292003; Q008: Quila 292008; Q009: Quila 292009; Q010: Quila 292010; Q011: Quila 292011; Q012: Quila 292012; Q013: Quila 292013; Q014: Quila 292014; Q015: Quila 292015; Q017: Quila 292017; Q018: Quila 292018; Q901: Quila 297901; Q101: Quila 279101; Q801: Quila 299801; Q802: Quila 299802; Q803: Quila 299803. White rice cultivar: Zafiro.

**Figure 4:**
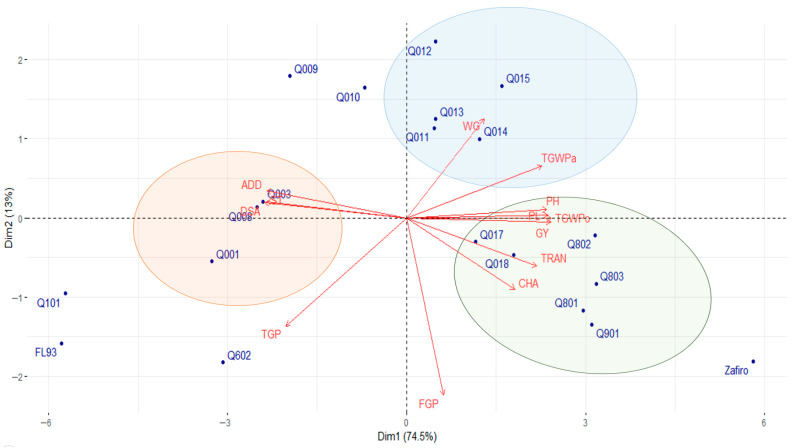
Principal components analysis (PCA) biplot of agronomic, productive, and quality traits of nineteen black rice genotypes and a white rice cultivar assessed under NFI conditions over three consecutive years. GY: grain yield; PH: plant height; DSA: days from sowing to anthesis; WG: whole grain percentage, CHA: grain chalkiness, TRA: grain translucency, PL: panicle length, TGP: total grain per panicle, FGP: filled grain per panicle; ST: sterility percentage; TGWPa: thousand-grain weight of paddy rice; TGWPo: thousand-grain weight of polished rice; ADD: average degree of dispersion. Black rice genotypes; FL93: FQuila; 93; Q602: Quila 291602; Q001: Quila 292001; Q003: Quila 292003; Q008: Quila 292008; Q009: Quila 292009; Q010: Quila 292010; Q011: Quila 292011; Q012: Quila 292012; Q013: Quila 292013; Q014: Quila 292014; Q015: Quila 292015; Q017: Quila 292017; Q018: Quila 292018; Q901: Quila 297901; Q101: Quila 279101; Q801: Quila 299801; Q802: Quila 299802; Q803: Quila 299803. White rice cultivar: Zafiro.

Principal component analysis (PCA) was conducted to establish the relationships between genotypes and agronomic, productive, and quality traits evaluated under different irrigation methods (Figure 3 and Figure 4). The PCA for flooding conditions explained 92.4% of the total variance, with key characteristics such as DSA, FGP, ADD, ST, TGWPo, PL, PH, TGWPa, and TGP accounting for 50% of the variability (Figure 3). The black rice genotypes were grouped into three distinct clusters, classifying Zafiro as a separate genotype. Among the pigmented rice genotypes, Quila 292001, Quila 292003, Quila 2912010, and Quila 291602 were closely associated with TRAN. Genotypes Quila 292008, Quila 292009, Quila 292011, and Quila 292012 formed a cluster linked to ADD, ST, PH, and DSA. The third cluster consisted of lines Quila 292014, Quila 292015, Quila 292017, Quila 292018, Quila 299801, Quila 299802, and Quila 299803. It was strongly associated with productivity-associated traits, including WG, PL, TGWPa, TGP, FGP, GY, and TGWPo.

The PCA under NFI conditions revealed a different grouping pattern than those observed under flooding (Figure 4). This analysis explained 87.5% of the variance in data using the first two principal components. The traits TGWPo, GY, DSA, PL, PH, ST, ADD, TGWPa, and TRAN accounted for 50% of the variability. Similarly, three distinct clusters were identified, with the white rice cultivar Zafiro being separated from all groups. The black rice genotypes Quila 292011, Quila 292012, Quila 292013, Quila 292014, and Quila 292015 were closely grouped and associated with WG and TGWPa. ADD and DSA primarily influenced lines Quila 292001, Quila 292003, and Quila 292008. Finally, genotypes Quila 292017, Quila 292018, Quila 299801, Quila 299802, Quila 299803, and Quila 297901 formed a separate cluster strongly linked to PH, PL, TGWPo, GY, TRAN, and CHA (Table S2).

Regarding the association between traits and environments, DSA, WG, and ST showed strong correlations (Figure S4) and were linked to NFI conditions. In contrast, PH, TGP, TGWPa, and TGWPo also exhibited a high correlation. However, their relationship was opposite to that of DSA, WG, and ST, resulting in a negative correlation between the two trait groups. The flooding conditions for 2021 and 2023 were closely clustered, suggesting similar effects influenced by FGP and GY. However, the association with these traits was not observed in 2022.

### 2.4. Genotypic and Phenotypic Correlations Among Traits

Genotypic correlations between yield and the other evaluated traits were generally low, except for the correlation with ST (−0.84) (Table 3). Furthermore, GY showed positive correlations (r = 0.46–0.57) with DSA, FGP, and TGWPa, as well as with the grain quality traits TRAN and CHA, with correlation coefficients of 0.48 and 0.59, respectively. DSA was positively correlated with ADD (0.72) and negatively correlated with ST (−0.53). The ST also correlated negatively (r = −0.51– −0.67) with FGP, TGWPa, and TGWPo, as well as with CHA and TRAN, with regression values of −0.64 and −0.73, respectively (Table 3).

Grain weight showed positive correlations (0.61–0.82) with grain quality traits (TRA, CHA, and WG) and negative correlations with ST, specifically −0.67 and −0.61 for TGWPa and TGWPo, respectively. Additionally, TRA and CHA were negatively correlated with ST, with values of −0.73 and −0.74, respectively. Considerably high correlations were found between TRA and CHA (0.98) and TGP and FGP (0.85). Phenotypic correlations generally followed a similar pattern, exhibiting comparable or lower values than genetic correlations.

### 2.5. Variance Components and Heritability of Traits

The combined analysis of variance revealed significant effects of genotype, environment, and genotype x environment interaction (Table 4). The genotypic variance was statistically significant for all traits evaluated except for ST (*p* < 0.05). Similarly, environmental variance was important for all traits except for CHA. Genotype x environment interaction variance was insignificant for DSA, PL, FGP, and TGP. Several traits exhibited very high heritability (H^2^ > 0.9), including grain weight (TGWPa and TGWPo), quality traits (CHA, TRA, and ADD), as well as DSA and PH. GY demonstrated moderate heritability (0.76). Instead, WG showed moderately high heritability. The lowest heritability values were observed for ST and PL, with values of 0.46 and 0.51, respectively (Table 4).

## 3. Discussion

### 3.1. Genotypic and Environmental Influences on Agronomic Performance and Grain Quality

Rice (*Oryza sativa* L.) is one of the most important staple crops worldwide, feeding more than half the global population. The agronomic performance of this crop is mainly influenced by genetic factors [14,42,43]. However, environmental conditions such as water availability, temperature fluctuations, and soil properties are critical in determining the final yield and quality of the crop. Parameters like the number of days to flowering play a crucial role in rice performance, particularly in Mediterranean climates like Chile, where water becomes limited during the reproductive phase. In these environments, earlier-flowering genotypes may hold a relative advantage. On average, black rice genotypes flowered about 8 days earlier than the white rice cultivar (Zafiro) (Table 1). This earlier flowering, frequently associated with shorter vegetative growth, helps avoid terminal stress but often results in lower biomass accumulation and yield potential [44,45,46]. In addition, pigmented rice genotypes tend to show reduced productivity compared to white rice cultivars, which explains why farmers in various regions are often reluctant to cultivate pigmented rice varieties [44,45]. These results also highlight the genetic differences that exist between traditional white rice and pigmented varieties.

Several studies have evidenced the lower yield stability of black rice genotypes across different environments [45,47,48], largely due to high sterility rates and reduced grain weight. Limbongan et al. [49] reported that the highest-yielding white rice genotype, UKIT102-2-056, produced 8.3 t ha^−1^; in contrast, the black rice genotype, UKIT104-2-127, reached a significantly lower yield of 3.4 t ha^−1^ under the same environmental conditions. In addition, the authors emphasized the importance of breeding programs in improving sterility resistance in pigmented rice varieties and enhancing the economic viability of the crop.

In the present study, the thousand-grain weight of the pigmented genotypes was lower than that of Zafiro, providing further evidence that black rice genotypes have lower yields than white rice. The reduced yield of black genotypes can be attributed to a high percentage of floral sterility. In contrast, this lower sterility rate in Zafiro being a temperate *O. sativa* subsp. *japonica* cultivar which is adapted to cooler temperatures [26,50]. Zafiro was the first cultivar developed in Chile through population improvement with recurrent selection, with cold tolerance as the primary target. It is particularly well-suited to the local conditions of Chile, the southernmost rice-growing region in the world. However, Quila 292001 and FLQuila 93 showed low sterility rates compared to other pigmented genotypes, which could indicate relatively higher cold tolerance among the pigmented rice genotypes.

In terms of grain quality, slight differences were found between pigmented genotypes and the white rice cultivar, particularly in WG, which is the main industrial quality parameter [51] and in the ADD, a measure of grain gelatinization temperature [18,52,53]. These shifts in WG and ADD suggest that while the visual attributes of pigmented rice differ substantially, its basic industrial processing characteristics might not be as drastically altered compared to white rice. As expected, significant differences were observed in grain appearance, with notable reductions in CHA and TRAN in pigmented genotypes. This highlights the visual distinction of pigmented varieties from white cultivars, which is a primary characteristic that differentiates them and is relevant to consumer preferences and market niches.

Concerning environmental factors, water status is one of the most critical determinants of rice growth, development, and yield, and thus, proper water management is crucial in the cultivation of this crop [26,27,28,49]. Traditional flooding irrigation has been the standard method in many rice-producing regions; however, this type of irrigation results in rice production with a high volume of water, which poses a problem in the face of increasing water scarcity. Therefore, alternative irrigation strategies, such as NFI, have been introduced [14,54].

This delay in the reproductive transition would indicate that this water-saving technique produces an alteration in the physiological processes that control growth and, therefore, has repercussions on grain formation and yield. Additionally, plant height decreased by 7.6%, a response to water deficit that affects cell elongation and internode expansion [28], while the most significant impact of NFI was a substantial decrease in grain yield, which fell by 28% compared to the conventional irrigation method (Table 1). This yield reduction was primarily attributed to a 12.4% increase in floral sterility and a 7.6% decline in filled grains per panicle. The increased sterility suggests that water limitations during reproductive development disrupt successful pollination and grain filling, leading to a higher proportion of unfilled grains [28]. However, grain weight was relatively unaffected, with only a reduction of approximately 2% (Table 1), suggesting grain weight stability and strong genetic control of this trait [34].

Despite its adverse effects on productivity, NFI had a minimal impact on grain quality traits such as CHA and TRAN, while ADD decreased. This indicates high heritability and stability across both water management conditions (Table 2). The CHA is primarily influenced by genetic factors that regulate starch biosynthesis and the grain-filling process. Genes involved in starch synthesis, such as SSIIa (starch synthase IIa) and Wx (waxy gene), play a key role in determining the structure of endosperm starch, which directly affects grain opacity [55]. The stability of CHA under NFI suggests that the expression of genes involved in starch biosynthesis is not highly sensitive to moderate variations in water availability, thus preserving the integrity of endosperm development. Similarly, TRAN, which is linked to the uniform packing of starch granules and the protein matrix within the endosperm, is primarily regulated by genetic factors [56]. The lack of significant variation in TRAN under NFI conditions indicates that water stress does not substantially disrupt the deposition of amylose and amylopectin or the protein-starch interaction within the grain. The ADD reflects the gelatinization temperature of rice starch, which is closely related to the fine structure of amylopectin. Genetic factors, such as the Wx locus, regulate both amylose content and gelatinization temperature [52]. The results suggest that the use of NFI does not significantly impact amylose content or starch molecular structure, resulting in a stable ADD under both flooding and NFI conditions. Regarding WG, the NFI resulted in a decline of 8.4%. This reduction could be due to a low translocation of assimilates, which alters the regular grain filling process, resulting in empty or immature grains that possess low milling strength and are prone to breakage during post-harvest processing [52].

In general, these findings suggest that NFI can significantly affect yield-related traits due to its effect on reproductive development and grain filling. However, this condition does not substantially alter the biochemical pathways that regulate grain quality traits. A study by Hallajian et al. [41] found that NFI could be an effective water management strategy, as it improves drought resistance. Nevertheless, they emphasized the need for genetic improvements to enhance sterility resistance and prevent yield reductions. Therefore, the development of black rice genotypes with enhanced performance under variable irrigation conditions could make NFI a viable water-saving strategy that preserves grain quality and supports effective postharvest processing and market acceptance.

Temperatures during the reproductive stage are particularly critical in rice cultivation, as they directly influence flowering, fertilization, and grain filling processes [14,22,57]. Heat stress exceeding 35 °C can disrupt anther dehiscence and pollen viability, leading to incomplete fertilization and spikelet sterility, which ultimately reduces grain yields [58]. These effects are further exacerbated when water deficit coincides with this stage, as limited transpiration restricts canopy cooling, thereby exposing reproductive tissues to additional thermal stress [22]. Rice genotypes differ in their ability to tolerate these combined stresses, depending on the expression of adaptive traits related to pollen fertility and heat tolerance [58].

During the three growing seasons (2021, 2022, and 2023), analysis of the critical thermal window revealed distinct climatic patterns between flooded and NFI regimes, with significant implications for reproductive performance. Under flooded conditions in 2021, daily mean temperatures generally remained within optimal ranges (20–30 °C); however, 61 days during sowing and tillering registered minimum temperatures below 10 °C, indicating potential cold stress during germination (Tables S4 and S5). During flowering, 14–16 days exceeded 30 °C, and six sequences of high daily thermal amplitude (ΔT > 18 °C) suggested transient heat stress episodes that could have reduced pollen viability and grain set efficiency (Table S4).

In 2022, flooded trials again experienced low temperatures at sowing, with up to 59 days below 12 °C, but also showed an increase in heat episodes during anthesis, with maximum temperatures above 33 °C recorded on several consecutive days. These conditions coincided with a reduction in fertile spikelets and contributed to asynchronous panicle emergence, consistent with the observed decline in grain yield and increased sterility rates (Table S5).

Under NFI management, the 2021 and 2022 seasons experienced relatively moderate microclimatic conditions, with fewer extreme events. However, during 2023, there was a marked increase in warm days and extended periods of high thermal amplitude, with up to seven consecutive days exceeding ΔT > 18 °C, particularly during grain filling (February –April (Table S4). Although average temperatures remained within optimal thresholds, repeated exposure to maximum temperatures above 33 °C and minimums below 22 °C during the reproductive stage likely imposed moderate cumulative thermal stress, negatively affecting pollen dehiscence and the uniformity of grain filling (Table S5).

Collectively, these results indicate that the 2022 season was the most thermally unstable, characterized by low early-season temperatures, accelerated phenology, and heat episodes during anthesis and grain filling. This pattern corresponds with the observed reduction in accumulated degree days and the shortened duration to heading reported for that year. In this season, rice grain yield declined sharply, and spikelet sterility increased both under flooded and NFI conditions These reductions were attributed to unfavorable climatic conditions, including low temperatures during germination, insufficient rainfall, and high temperatures during the reproductive stage (January to March) (Figure S1). The concurrence of cold stress during crop establishment and heat stress during reproduction supports the hypothesis that temperature fluctuations, rather than absolute extremes, were the primary climatic driver of spikelet sterility and yield reduction. A correlation analysis between DSA and accumulated degree days revealed that the conditions described above had a negative impact, particularly during the 2022 season (Figure S2). Specifically, a difference in the DSA was observed between years with flooding, with 18 fewer days in the 2022 season than in the average of the other two seasons. This shortened developmental window likely led to asynchronous panicle emergence and reduced pollen viability under heat and limited water availability, resulting in fewer fertile spikelets and fewer effective panicles per unit area. The combination of these stresses consequently reduced yield [22], which underscores the need to develop genotypes with enhanced thermotolerance and reproductive resilience under contrasting hydrological regimes in the Chilean rice-growing regions. Consequently, the combined effects resulted in significant yield losses, making the 2022 season particularly challenging for the Chilean rice industry [59]. Therefore, these findings underscore the importance of investigating the mechanisms of stress adaptation, thereby facilitating the selection of genotypes suited to varying environmental conditions [60].

### 3.2. Correlation Among Agronomic, Productive, and Quality Traits

Understanding the correlation between grain yield and agronomic, productive, and quality traits is crucial for rice breeding as grain yield is a complex trait influenced by multiple genes, while plant phenotype expression is also influenced by the environment [61,62]. Under NFI conditions, GY was highly correlated with most of the evaluated traits (Figure 2), albeit with some differences in the effect observed compared to conventional flooding.

As expected, grain yield under both conditions was primarily influenced by DSA, which positively affected ST but negatively impacted yield components such as PL and grain weight (Figure 2 and Figure 3). This pattern is well-documented, with early-flowering genotypes typically allocating more assimilates to reproductive structures, resulting in shorter plant height and enhanced grain-filling efficiency [63]. Early flowering has been recognized in crops as an escape mechanism under water stress conditions [54,64]. However, rice cultivars often delay flowering time [65,66], negatively impacting grain yield. This effect is particularly evident in the strong correlation between GY and DSA under NFI conditions (r = −0.99; Figure 2). Research on photoperiod-sensitive rice varieties reveals that flowering delays under stress conditions frequently result in yield losses, primarily due to a shorter grain-filling period and increased sterility [66]. Temperature fluctuations further exacerbate this issue by negatively affecting spikelet fertility [54].

Despite the apparent negative correlation between GY and DSA under both water irrigation methods, the association between GY and PH and between GY and TGP differed. It has been reported that an increase in plant height generally leads to higher yields in rice [63]. In the present study, however, this effect was observed only under NFI (Figure 2), where a strong positive correlation (r = 0.97) was found between GY and PH. The delayed flowering observed under NFI is likely a result of physiological stress responses [22], which can hinder carbohydrate translocation, ultimately affecting grain yield [65], thus leading to smaller and less productive plants.

Water stress and temperature fluctuations in rice have been shown to lead to floral sterility, a reduction in the number of mature pollen grains, and decreases in the number of effective panicles, grains per panicle, and grain yield [54,57,67]. The decline in effective panicles and grain set under NFI conditions may be linked to oxidative stress, as elevated reactive oxygen species (ROS) impair pollen viability and stigma receptivity [54]. This could result in a non-significant correlation between GY and FGP.

The low correlation values of CHA under flooding conditions suggest that this trait is not associated with agronomic and yield characteristics, except for TRAN, which showed a significant positive correlation with CHA. This results in a translucent grain grade, a highly valued characteristic in the rice market [68], significant in premium rice markets of japonica varieties [14]. A different trend was observed under NFI, with CHA showing substantial correlations with most of the evaluated traits. This suggests a water stress effect, even though genetic factors primarily influence CHA [55] and remain consistent across genotypes and environments. On the other hand, the correlation between TRAN and GY demonstrated contrasting effects across different environments. A negative association was observed under flooded conditions, while a positive association was noted under NFI (Figure 1 and Figure 2). This pattern persisted regardless of the slight impact of NFI on grain translucency, although NFI did significantly affect grain yield (Table 1 and Table 2).

### 3.3. Principal Component Analysis of Trait Variation

Principal component analysis (PCA) was performed to explore the relationships between genotypes and agronomic, productive, and quality traits under the different irrigation methods, thereby facilitating an understanding of the underlying data structure [69,70].

A clear positive correlation was observed between grain yield and traits such as PL, TGP, FGP, and grain weight (Figure 3), which are key determinants of crop productivity [14]. These traits are susceptible to environmental conditions since optimal water availability is required for panicle initiation, flowering, and grain development [22,71]. In rice, flooding enhances vegetative growth and reproductive success by sustaining optimal transpiration rates and preventing spikelet sterility [72,73]. However, the present study observed negative associations between GY and DSA, ST, and PH. Delayed flowering in rice typically results in reduced grain yield [65,66], while elevated ST is also linked to lower productivity, particularly in temperate climates, where temperature fluctuations during the reproductive stages can disrupt flowering and diminish crop yield [14,22,57].

As mentioned above, water stress in rice leads to altered carbohydrate metabolism, which negatively affects grain development [54,57,74] and, therefore, grain weight. These results suggest that genotypes under water deficit conditions, which achieve higher biomass production—evident from the positive association between GY with PH and PL—have a greater capacity to achieve grain filling and, consequently, higher yields. As observed under flooded conditions, GY was negatively correlated with DSA and ST (Figure 4) since delayed flowering negatively impacts yield by shortening the grain-filling period and increasing floral sterility [66]. In contrast, under non-flooded conditions, the absence of the thermoregulatory effect of water, combined with low nighttime or high daytime temperatures, adversely affects spikelet fertility [54]. Additionally, Quila 299801, Quila 299802, and Quila 299803 under NFI conditions exhibited relatively high CHA and TRAN values, highlighting a positive correlation between these quality traits and grain yield. Water stress and high temperatures are also known to affect grain quality in rice negatively [54,75]. Reduced water availability during critical grain-filling stages led to incomplete and irregular starch granule formation, which resulted in lower grain quality [14,18]. These results are consistent with previous studies in which chalkiness and translucency were affected under NFI by altering amylose content and protein-starch interactions [19,52]. In contrast, genotypes Quila 292001, Quila 292003, and Quila 292008 form a cluster primarily influenced by DSA, ST, and ADD (Figure 4). Under NFI conditions, these genotypes exhibited yields lower than the average of the black genotypes, with DSA being higher and ADD being like or higher than the average value of the black genotypes (Table S2). This highlights the relationship between water stress in rice and its impact on grain quality, affecting not only chalkiness and translucency but also gelatinization temperature (i.e., ADD) [14,18,19]. Finally, a third cluster, primarily defined by WG and, to a lesser extent, by TGWPa, grouped genotypes Quila 292011, Quila 292012, Quila 292013, Quila 292014, and Quila 292015. This cluster illustrates the positive association between WG and TGWPa, although WG showed only a slight and non-significant correlation with GY under NFI conditions (Figure 2).

The black genotypes Quila 279101 and FQuila 93, and the white genotype Zafiro, were the most responsive based on the evaluated traits in both environments, flooding and NFI (Figure 3 and Figure 4). These genotypes exhibited distinct performances in both environments compared to the other genotypes and were not grouped in any cluster. Under flooded conditions, Zafiro exhibited the highest values for DSA, GY, grain weight, CHA, and TRAN, while it tended to have the lowest values for ST. On the other hand, although FQuila 93 had a particularly low yield, it tended to have the highest WG value. While Quila 279101 had a high yield among the pigmented genotypes, it also tended to show the highest TGP; however, it reached a relatively high ST, while it tended to exhibit the lowest TWGPo, along with a high ADD value (Table S1). Likewise, similar trends were observed in the performance of these genotypes under NFI conditions (Table S2).

### 3.4. Genotypic and Phenotypic Correlations Among Traits

Information on the variability and heritable traits of the genetic material is crucial for effective yield and grain quality improvement in rice breeding. Understanding the phenotypic and genotypic components of variation, heritability, and correlations among traits provides valuable insights into breeding desirable traits [61,76].

If two traits are genetically positively correlated and selection favors high values for both traits, an evolutionary response is observed [77]. Genotypic correlations between grain yield and the other evaluated traits were generally low, except for the correlation with ST (r = −0.84) (Table 3). The positive correlation between yield and grain quality variables has been similarly described by Tiwari et al. [76] reported strong positive genotypic and phenotypic correlations between grain yield and both days to heading and days to maturity in rice under a rainfed lowland environment. In addition, significant positive genotypic and phenotypic correlations were obtained between GY and grain weight, which agrees with that reported for elite rice lines developed for tropical lowland ecosystems [48].

Conversely, ST exhibited a negative correlation with grain yield and quality traits (Table 3). These findings are consistent with those of Akbar et al. [27], who observed a negative correlation between grain sterility percentage and grain yield and quality traits in double haploid lines of lowland rainfed rice. This suggests that an increase in sterility percentage directly reduces yield, highlighting the importance of selecting genotypes with low sterility, especially under water stress and suboptimal conditions. Conversely, PH exhibited a positive and moderate genetic correlation with grain weight, with values of 0.50 for both TGWPa and TGWPo, contrasting with the findings of Tiwari et al. [76].

On the other hand, although no significant, negative genetic correlations were observed between TGP and grain weight (Table 3). It has been reported that genotypes with a high number of filled grains per panicle exhibit reduced grain weight, likely due to a limited assimilate supply [74], which aligns with the patterns observed in the present study. This phenomenon, often referred to as the “trade-off effect,” has been well-documented in cereal crops [78,79]. The trade-off occurs due to limited assimilate availability during grain filling, where an increase in grain number leads to competition for resources, ultimately reducing the individual grain weight [79]. These findings suggest that breeding programs should focus on selecting genotypes with optimal panicle architecture to balance grain number and grain weight.

The strong genotypic and phenotypic correlation between CHA and TRA underscores a close genetic relationship between these key grain quality traits. However, an evident pattern was observed in which the genetic correlation between grain yield and WG was not found to be positive, which is particularly relevant for genetic improvement strategies, since WG is a fundamental industrial quality trait; its reduction through grain breakage significantly decreases its market value [14,80]. Similarly, ADD, which influences gelatinization temperature and cooking behavior, is crucial for determining the technological and market characteristics of rice [18,81]. The absence of a genetic correlation between yield and these two industrial traits suggested a potential trade-off in selection efforts to improve productivity and processing quality in black rice.

### 3.5. Variance Components and Heritability

Heritability is a predictive function that measures genetic variability, especially in crop improvement, reflecting the effectiveness of genotype selection [61,82]. This index provides valuable information on genetic and environmental factors influencing trait expression in rice breeding programs [83]. High to moderate values of broad-sense heritability (H^2^ > 0.6) indicate strong genetic control over a trait, with a lesser impact of environmental factors [84].

Differences in genetic variances were found in all traits evaluated, except for ST, indicating the presence of genetic variations within the population [39]. In contrast, the variance of the genotype x environment interaction was not significant in some traits, suggesting their stability across the three years under different water regimes [13,84]. Similar results were reported by Tiwari et al. [76] for early maturing rice in rainfed lowland environments, with broad-sense heritability values of 0.94 and 0.92 for days to heading and days to maturity, respectively, and 0.74 for thousand-grain weight (TGW). Using a panel of 100 rice genotypes, Asante et al. [85] also reported H^2^ values of 0.81 for days to flowering and 0.62 for plant height.

The GY exhibited high heritability, as this parameter is a quantitative trait influenced by the combined effects of multiple genes (polygenic inheritance) and environmental factors, such as soil fertility, water availability, and temperature fluctuations [43,71]. In this study, environmental variability accounted for 24% of the yield variation, with NFI significantly reducing productivity. Similarly, Li et al. [79] observed that high environmental variation in grain yield was associated with decreased productivity due to water stress. On the other hand, Asante et al. [85] and Adjah et al. [40] reported low heritability for grain yield (0.23 and 0.37, respectively) when using a larger rice accessions pool and under traditional flooded conditions, where greater genetic diversity could result in higher variability in rice performance.

Grain quality traits showed moderate to high heritability, indicating genetic control. Previous reports have shown relatively high H^2^ values for percentage of chalky grains and the degree of chalkiness, reaching 0.69 and 0.66, respectively, and a low value of 0.15 for the percentage of broken rice grains [40]. The high heritability observed in this study agrees with Demeke et al. [86], who demonstrated that selecting for high-heritability traits such as days to heading, maturity, plant height, and thousand-grain weight effectively improves yield across environments. In addition, the PCAs (Figure 3 and Figure 4) further highlighted that those high-heritability traits are key contributors to yield variance across different environments [87].

The correlations between agronomic and quality traits provide a strong basis for indirect selection in breeding programs. High heritability values for traits such as TGWPa, TGWPo, TRAN, and CHA (Table 4) indicate that these traits are predominantly influenced by genetic factors, making them reliable targets for genetic improvement in black rice to enhance both yield stability and grain quality under water-saving irrigation. Integrating multi-environment trials and genomic prediction models (e.g., genome-wide association studies (GWAS) could enhance selection efficiency by identifying stable genotypes with desirable trait combinations [88].

## 4. Materials and Methods

### 4.1. Plant Material, Experimental Design, and Crop Management

**Figure 5:**
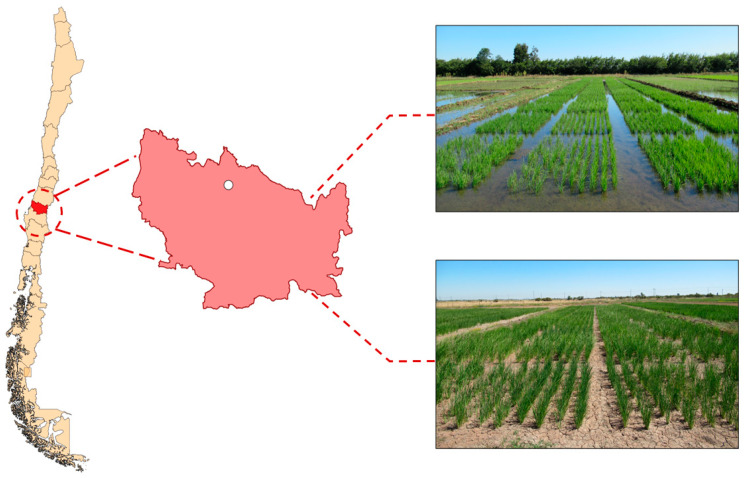
Location of the San Carlos site in the Ñuble Region, and experimental plots under traditional flooding (**top**) and NFI (**bottom**).

Nineteen advanced black rice genotypes and the white rice cultivar Zafiro, developed by the Rice Breeding Program (RBP) of the National Institute of Agricultural Research (INIA), Chile, were evaluated. Genotypes were selected based on their performance in a preliminary screening of black rice lines from the RBP, with an emphasis on grain color and the stability of this trait. On the other hand, Zafiro, which accounts for approximately 70% of the rice cultivated area in Chile, was included as a control genotype. The trial was conducted in the INIA experimental rice fields in San Carlos (36°25′49″ S, 72°0′25″ W; 161 m.a.s.l.), Ñuble region. The germplasm was evaluated over three years (2021, 2022, and 2023) under two water regimes: Flooding and NFI (Figure 5).

In the NFI treatment, irrigation was applied to maintain 35% of available water content in soil, as measured using a Time Domain Reflectometry (TDR) sensor (Campbell Scientific, Logan, UT, USA). Irrigation intervals averaged 12 days during the early growth stages and shortened to approximately 8 days from late November onward, coinciding with tiller initiation and rising air temperatures. This schedule was maintained throughout the growth cycle, except during the period between tillering and flowering, when a layer of water was applied to prevent damage to flower production. In contrast, the flooded control was maintained under a 10 cm layer of water, with continuous replenishment throughout the growth cycle. Water consumption under this system was estimated at 21,000 m^3^ ha^−1^, whereas total water use under NFI was reduced to 11,700 m^3^ ha^−1^, representing a 55.7% saving.

A completely randomized block design with three replicates (blocks) was used. Each plot consisted of seven 3 m long rows spaced 0.3 m apart, resulting in a plot area of 6.3 square meters. Planting/sowing date and harvest date are shown in Table S3. In traditional flooded cultivation, the planting density was 140 kg ha^−1^. To achieve a comparable stem density under both irrigation systems, the planting density was reduced to 40 kg ha^−1^ in the NFI treatment. Previous studies under similar conditions have shown that plants produce an average of 15 tillers under NFI, compared to 3–5 under the conventional flooded system, as the absence of a water table promotes greater tillering and a more extensive root system [89]. The soil corresponds to the Tiuquilemu series, classified as fine loamy, mixed, superactive, thermic Fluventic Xerochrepts. It features flat topography, moderate permeability, and a parent material of fluvial deposits over a volcanic tuff substrate [90]. Prior to the trial, the field was always left fallow. Conventional soil management practices were implemented before trial establishment, including chemical fallowing with glyphosate (2 L ha^−1^), leveling, and soil preparation. For optimal plant growth, NPK fertilizer was applied before sowing. Urea (CH_4_N_2_O) was used at a rate of 22 kg ha^−1^, triple superphosphate (Ca(H_2_PO_4_)_2_H_2_O) at 130 kg ha^−1^, and muriate of potash (KCl: NaCl) at 150 kg ha^−1^. Irrigation was initiated one day after planting, with subsequent water applications promoting seedling emergence and supporting plant establishment.

Once the plants reached the 2.5-leaf stage and until grain filling was complete, plants under the flooding regime remained flooded, while those under NFI were irrigated every eight days at field capacity. Nitrogen was applied in three additional phenological stages: 66 kg ha^−1^ of urea at the 2.5-leaf stage, 88 kg ha^−1^ at tillering, and 44 kg ha^−1^ at panicle initiation. Weed control was carried out using Ricer^®^ (210 cc ha^−1^) and Clincher^®^ (1.5 L ha^−1^) to manage narrow-leafed weeds and Bentax (2.5 L ha^−1^) and MCPA (1 L ha^−1^) for controlling wide-leafed weeds.

### 4.2. Assessment of Agronomic, Productive, and Quality Traits

Days from sowing to anthesis (DSA) and plant height (PH) were recorded at anthesis. At harvest, panicle length (PL), total number of grains per panicle (TGP), number of filled grains per panicle (FGP), panicle sterility percentage (ST), thousand-grain weight of polished (TGWPo) and paddy (TGWPa) rice, and grain yield (GY) at 14% moisture content were evaluated.

To determine the whole grain percentage (WG), 100 grains were polished using a Suzuki MT test mill. Grain chalkiness (CHA) and translucency (TRAN) were measured using an MM1D milling meter (Satake, Japan), with three readings taken per sample. For the average degree of dispersion (ADD) in the alkali reaction, ten representative whole grains were incubated for 23 h at 30 °C in a 1.7% KOH solution, after which the gelatinization temperature was measured [21].

### 4.3. Linear Mixed Models and Genotype X Environment Interaction

Agronomic, productive, and quality traits were analyzed using linear mixed models (LMM), with water management and year combinations representing six distinct environments. The data were subjected to a combined analysis of variance to assess the genetic variability of rice genotypes across the environments, according to the following equation [38]:

$$Y_{ijk}=\mu +{Gen}_{i}+{Env}_{j}+{Rep}_{k}({Env}_{j})+({Gen}_{i}\;x\;{Env}_{j})+\varepsilon_{ijk}$$

$Y_{ijk}$ is the observation for the ith genotype in the kth replication in the jth environment (water management x year); μ is the gran mean; ${Gen}_{i}$ is the effect of the ith genotype considered as fixed; ${Env}_{j}$ is the effect of the jth environment considered as random; ${Rep}_{k}({Env}_{j})$ is the effect of the kth replication within the jth environment; $\left({Gen}_{i}\;x\;{Env}_{j}\right)$ is the random effect of the interaction between ith genotype with the jth environment; $\varepsilon_{ijk}$ is the random effect of the error associated with the ith genotype, jth environment and kth replication, which is supposed to be independent ε ~ N(0, σ2).

The broad-sense heritability based on the means in the multi-environment model was estimated as follows:

$${H\;}^{2}=\frac{\sigma_{g}^{2}}{\begin{array}{c} \sigma_{g}^{2}+\frac{\sigma_{ge}^{2}}{nEnv}+\frac{\sigma_{\varepsilon }^{2}}{\begin{array}{c} n\mathrm{Env}\;x\;n\mathrm{Rep} \end{array}} \end{array}}$$

where $\sigma_{g}^{2}$, $\sigma_{ge}^{2}$, and $\sigma_{\varepsilon }^{2}$ represent the variances of genotype, genotype x environment interaction, and error, respectively. nEnv and nRep denote the number of environments and replications, respectively.

The genetic correlation between traits was determined using the following equation:

$$\rho_{ց=\frac{\bar{\sigma_{g(\mathrm{jj}\prime )}}}{\bar{\sigma_{g(j)}\sigma_{g(j\prime )}}}}$$

where $\bar{\sigma_{g(\mathrm{jj}\prime )}}$ is the arithmetic mean of all pairwise genotypic variances between traits j and j′ and $\sigma_{g(j)}\sigma_{g(j^{\prime })}$ represents the arithmetic average of all pairwise means of the genotypic variance components for the traits.

### 4.4. Statistical Analysis

Statistical analysis, including the calculation of broad-sense heritability and genetic and phenotypic correlations, was performed using META-R (Multi-Environment Trial Analysis using R) software, version 6.0 [91]. Correlation and principal component analysis (PCA) were conducted in RStudio, version 4.2.1 [92].

## 5. Conclusions

This study offers new insights into the complex interplay between genetic factors, environmental variability, and water management strategies that influence yield and grain quality in black rice, which is particularly significant given the limited research on black rice performance under water-limited conditions and the scarcity of studies conducted in Mediterranean climates, such as those found in central-southern Chile.

The 2022 season was characterized by fluctuating temperatures during the early growth stages and flowering, along with lower accumulated degree days. These factors significantly affected plant performance, resulting in reduced yield, fewer filled grains per panicle, and increased panicle sterility (ST), particularly under flooded conditions.

The multi-environment analysis revealed significant genotypic and phenotypic correlations between yield and industrial-quality traits, along with high heritability for some agronomic and quality traits, indicating their potential for indirect selection of genotypes combining high yield and superior grain quality under water-saving irrigation. These traits thus represent valuable targets for black rice breeding programs focused on developing cultivars with enhanced resilience to environmental variability. In contrast, grain weight, plant height, and panicle length did not show significant correlations, whereas ST exhibited a strong negative correlation with yield, reflecting a plastic adaptive response.

The findings underscore the importance of an integrated approach that combines genetic selection, optimized agronomic practices, and strategies to enhance water use efficiency and maintain the sustainability of rice cultivation. It also highlights the importance of future research focusing on identifying genetic markers associated with stress tolerance and resistance to sterility by incorporating genomic selection and multi-environment testing, which could contribute to the development of high-yielding, high-quality, and climate-resilient black rice cultivars.

## Figures and Tables

**Table 1:** Analysis of variance of agronomic and productive traits evaluated in 19 black rice genotypes and the white cultivar Zafiro grown under six different environments resulting from the combination of water management (conventional flooding (F) and non-flooding irrigation (NFI)) and three years (2021, 2022 and 2023).

	DSA (Days)	PH (cm)	GY (t ha^−1^)	FGP	TGP	ST (%)	PL (cm)
Genotype (G)							
FLQuila 93	123.56 ± 3.17 ^bcde^	91.72 ± 2.36 ^ab^	3.82 ± 4.11 ^cde^	48.76 ± 2.24 ^abc^	79.80 ± 3.39 ^cde^	37.67 ± 2.98 ^de^	17.57 ± 0.30 ^fgh^
Quila 279101	125.33 ± 2.87 ^b^	85.17 ± 2.30 ^ab^	4.57 ± 5.14 ^abc^	49.50 ± 4.72 ^abc^	91.64 ± 4.39 ^ab^	45.82 ± 4.69 ^b^	18.98 ± 0.28 ^bc^
Quila 291602	123.56 ± 3.13 ^bcd^	90.19 ± 2.79 ^ab^	4.60 ± 7.20 ^bcd^	47.60 ± 3.20 ^bc^	82.12 ± 4.99 ^bcde^	40.71 ± 3.27 ^bcde^	16.27 ± 0.37 ^i^
Quila 292001	122.72 ± 2.95 ^bcde^	80.61 ± 2.33 ^a^	4.73 ± 6.28 ^abc^	49.98 ± 3.98 ^abc^	77.63 ± 4.79 ^def^	35.44 ± 3.64 ^ef^	17.57 ± 0.28 ^fgh^
Quila 292003	120.83 ± 3.39 ^bcde^	76.42 ± 3.22 ^ab^	4.01 ± 5.90 ^cde^	51.41 ± 3.69 ^abc^	86.58 ± 5.13 ^abcd^	38.94 ± 3.92 ^cde^	18.32 ± 0.98 ^fgh^
Quila 292008	123.50 ± 3.01 ^bcd^	74.39 ± 2.03 ^ab^	3.14 ± 4.46 ^e^	44.92 ± 2.33 ^cd^	78.72 ± 2.03 ^cdef^	42.18 ± 3.45 ^abcd^	17.17 ± 0.32 ^hi^
Quila 292009	123.28 ± 2.80 ^bcd^	73.64 ± 2.74 ^ab^	4.74 ± 6.25 ^ab^	38.89 ± 3.19 ^de^	69.52 ± 4.42 ^f^	44.60 ± 2.89 ^abc^	17.25 ± 0.29 ^hi^
Quila 292010	121.83 ± 2.93 ^bcdef^	81.94 ± 2.11 ^ab^	4.27 ± 5.75 ^bcd^	49.70 ± 4.52 ^abc^	87.76 ± 3.35 ^abc^	42.78 ± 4.84 ^abcd^	17.82 ± 0.24 ^efgh^
Quila 292011	124.33 ± 3.25 ^bc^	89.89 ± 2.59 ^b^	4.29 ± 6.17 ^bcd^	45.54 ± 3.50 ^cd^	82.65 ± 3.05 ^bcde^	44.94 ± 3.70 ^abc^	18.55 ± 0.25 ^bcd^
Quila 292012	121.78 ± 2.75 ^cdef^	80.08 ± 2.20 ^a^	3.25 ± 4.94 ^e^	35.36 ± 3.13 ^de^	68.93 ± 3.32 ^f^	48.15 ± 4.02 ^a^	19.89 ± 2.27 ^efg^
Quila 292013	121.11 ± 3.11 ^bcedf^	79.81 ± 2.63 ^ab^	4.14 ± 5.88 ^bcd^	51.00 ± 3.66 ^abc^	83.52 ± 3.42 ^abcde^	39.25 ± 3.20 ^bcde^	17.35 ± 0.25 ^gh^
Quila 292014	123.44 ± 2.98 ^bcd^	84.44 ± 2.56 ^ab^	4.20 ± 5.68 ^bcd^	46.24 ± 3.15 ^cd^	85.93 ± 3.04 ^abcd^	45.15 ± 4.20 ^abc^	18.01 ± 0.28 ^def^
Quila 292015	122.06 ± 3.24 ^bcde^	81.81 ± 1.97 ^ab^	4.41 ± 6.03 ^abcd^	44.19 ± 3.76 ^cd^	75.56 ± 4.77 ^ef^	41.71 ± 3.14 ^abcde^	17.88 ± 0.22 ^efg^
Quila 292017	118.94 ± 3.10 ^ef^	76.47 ± 2.41 ^ab^	4.18 ± 4.80 ^bcd^	56.06 ± 5.16 ^a^	93.50 ± 4.50 ^a^	40.71 ± 4.46 ^bcde^	17.19 ± 0.41 ^fgh^
Quila 292018	120.67 ± 3.11 ^def^	81.06 ± 2.34 ^ab^	3.53 ± 4.96 ^de^	48.11 ± 3.91 ^abc^	82.99 ± 3.03 ^bcde^	42.39 ± 3.68 ^abcd^	18.07 ± 0.27 ^def^
Quila 297901	110.00 ± 3.02 ^g^	83.89 ± 2.53 ^ab^	3.81 ± 5.82 ^cde^	48.52 ± 3.91 ^abc^	87.12 ± 2.98 ^abcd^	45.08 ± 3.51 ^abc^	17.54 ± 0.36 ^fgh^
Quila 299801	116.33 ± 2.59 ^f^	100.61 ± 2.33 ^b^	4.17 ± 3.76 ^abcd^	45.03 ± 3.12 ^cd^	77.23 ± 3.86 ^def^	41.11 ± 3.35 ^bcde^	18.34 ± 0.66 ^defg^
Quila 299802	123.44 ± 2.96 ^bcd^	84.36 ± 2.53 ^a^	4.94 ± 5.73 ^ab^	50.16 ± 4.16 ^abc^	82.21 ± 3.40 ^bcde^	39.51 ± 4.18 ^bcde^	19.65 ± 0.26 ^a^
Quila 299803	121.78 ± 3.22 ^bcde^	88.08 ± 2.03 ^ab^	4.46 ± 4.62 ^abc^	52.17 ± 4.78 ^abc^	87.18 ± 4.67 ^abcd^	40.90 ± 3.65 ^bcde^	19.11 ± 0.24 ^ab^
Zafiro	129.11 ± 3.02 ^a^	88.78 ± 2.65 ^ab^	6.04 ± 8.90 ^a^	54.47 ± 3.44 ^ab^	77.76 ± 1.76 ^cdef^	30.28 ± 3.73 ^f^	18.39 ± 0.24 ^cde^
Environment (E)							
F2021	120.13 ± 0.77 d	95.25 ± 1.50 ^a^	7.12 ± 2.38 ^a^	54.49 ± 1.40 ^b^	80.63 ± 1.19 ^bc^	32.46 ± 1.40 ^e^	16.92 ± 0.19 ^e^
F2022	116.15 ± 0.60 ^e^	90.78 ± 0.96 ^a^	3.62 ± 1.57 ^b^	41.26 ± 2.02 ^cd^	96.03 ± 2.26 ^a^	57.73 ± 1.62 ^a^	19.97 ± 0.67 ^a^
F2023	98.85 ± 0.50 ^f^	87.86 ± 1.01 ^a^	7.37 ± 1.93 ^a^	67.72 ± 1.58 ^a^	85.74 ± 1.57 ^b^	20.94 ± 1.21 ^f^	17.48 ± 0.24 ^d^
NFI2021	135.75 ± 0.71 ^a^	74.54 ± 1.46 ^a^	2.03 ± 1.24 ^c^	42.08 ± 1.22 ^cd^	78.93 ± 1.24 ^c^	46.28 ± 1.53 ^c^	17.06 ± 0.15 ^de^
NFI2022	133.72 ± 0.64 ^b^	73.69 ± 0.89 ^a^	2.21 ± 0.75 ^c^	38.39 ± 1.23 ^d^	77.77 ± 1.76 ^cd^	50.45 ± 1.31 ^b^	18.12 ± 0.15 ^c^
NFI2023	126.68 ± 0.67 ^c^	79.89 ± 1.08 ^a^	3.25 ± 1.26 ^b^	43.32 ± 2.36 ^c^	72.41 ± 3.35 ^d^	40.33 ± 1.55 ^d^	18.71 ± 0.31 ^b^
*p*-value							
G	0.0000	0.6718	0.0002	0.0002	0.0000	0.0001	0.0000
E	0.0000	0.9996	0.0000	0.0000	0.0000	0.0000	0.0000
G x E	0.9653	1.0000	0.9969	0.1641	0.8199	0.0035	0.0023

DSA: days from sowing to anthesis; PH: plant height; GY: grain yield; FGP: filled grain per panicle; TGP: total grain per panicle; ST: sterility percentage; PL: panicle length. Different letters in the same column indicate statistical differences according to the LSD Fisher test (*p* ≤ 0.05).

**Table 2:** Analysis of variance of productive and quality traits evaluated in 19 black rice genotypes and the white cultivar Zafiro-INIA grown under six environments resulting from the combination of water management (conventional flooding (F) and non-flooding irrigation (NFI)) and three years (2021, 2022 and 2023).

	WG (%)	TGWPa (g)	TGWPo (g)	CHA	TRAN	ADD
Genotype (G)						
FLQuila 93	63.36 ± 0.52 ^ab^	30.44 ± 0.51 ^bcd^	22.26 ± 0.32 ^c^	22.89 ± 0.92 ^g^	1.55 ± 0.10 ^ghi^	5.88 ± 0.15
Quila 279101	52.53 ± 3.11 ^abc^	27.13 ± 0.65 ^ghi^	18.21 ± 0.37 ^jk^	13.30 ± 0.89 ^j^	0.57 ± 0.06 ^k^	6.04 ± 0.18
Quila 291602	63.19 ± 1.53 ^a^	31.48 ± 0.45 ^b^	22.88 ± 0.37 ^b^	33.43 ± 0.44 ^b^	2.59 ± 0.06 ^b^	4.61 ± 0.21
Quila 292001	58.29 ± 2.26 ^abc^	29.02 ± 0.60 ^de^	20.34 ± 0.31 ^efg^	27.00 ± 0.70 ^cd^	1.84 ± 0.09 ^def^	6.01 ± 0.14
Quila 292003	59.10 ± 2.42 ^ab^	28.66 ± 0.71 ^ef^	20.52 ± 0.33 ^ef^	26.98 ± 0.74 ^cd^	1.87 ± 0.08 ^de^	5.92 ± 0.15
Quila 292008	58.90 ± 1.92 ^c^	25.79 ± 0.56 ^ij^	18.32 ± 0.41 ^jk^	18.09 ± 0.74 ^i^	0.96 ± 0.06 ^j^	5.71 ± 0.17
Quila 292009	63.72 ± 1.01 ^abc^	25.25 ± 0.49 ^j^	17.91 ± 0.30 ^k^	18.39 ± 0.79 ^i^	1.13 ± 0.11 ^hi^	5.97 ± 0.17
Quila 292010	58.94 ± 2.58 ^bc^	29.26 ± 1.48 ^cde^	19.94 ± 0.37 ^gh^	27.29 ± 0.67 ^cd^	1.84 ± 0.08 ^d^	5.91 ± 0.14
Quila 292011	60.88 ± 1.20 ^bc^	30.54 ± 0.52 ^bc^	21.98 ± 0.31 ^cd^	26.39 ± 0.75 ^cde^	1.89 ± 0.09 ^de^	5.87 ± 0.18
Quila 292012	62.64 ± 1.03 ^ab^	28.89 ± 0.58 ^e^	21.67 ± 0.40 ^d^	23.71 ± 0.96 ^fg^	1.54 ± 0.10 ^fgh^	5.76 ± 0.17
Quila 292013	57.90 ± 1.74 ^bc^	26.25 ± 0.39 ^hij^	18.63 ± 0.37 ^ij^	20.33 ± 0.84 ^h^	1.13 ± 0.07 ^ij^	5.54 ± 0.18
Quila 292014	60.29 ± 1.51 ^bc^	26.96 ± 0.51 ^ghi^	19.82 ± 0.33 ^gh^	25.90 ± 0.77 ^cde^	1.73 ± 0.08 ^de^	6.09 ± 0.17
Quila 292015	57.46 ± 2.09 ^bc^	27.08 ± 0.66 ^ghi^	19.98 ± 0.34 ^fgh^	25.64 ± 0.54 ^de^	1.67 ± 0.06 ^def^	5.82 ± 0.16
Quila 292017	54.46 ± 2.19 ^bc^	25.34 ± 0.74 ^j^	18.46 ± 0.31 ^jk^	22.74 ± 0.97 ^g^	1.26 ± 0.08 ^hi^	5.22 ± 0.24
Quila 292018	56.98 ± 1.81 ^bc^	25.89 ± 0.40 ^hij^	18.17 ± 0.32 ^jk^	20.88 ± 1.02 ^h^	1.13 ± 0.10 ^hij^	5.68 ± 0.18
Quila 297901	59.97 ± 1.45 ^ab^	28.35 ± 0.55 ^efg^	19.91 ± 0.35 ^gh^	32.16 ± 0.69 ^b^	2.26 ± 0.10 ^c^	2.93 ± 0.26
Quila 299801	57.38 ± 1.33 ^c^	27.27 ± 0.60 ^fgh^	19.67 ± 0.35 ^h^	26.43 ± 0.91 ^cde^	1.76 ± 0.11 ^efg^	5.73 ± 0.17
Quila 299802	62.37 ± 1.33 ^ab^	28.91 ± 0.38 ^e^	20.67 ± 0.30 ^e^	25.00 ± 1.01 ^ef^	1.61 ± 0.11 ^efg^	6.14 ± 0.17
Quila 299803	52.37 ± 2.21 ^c^	26.26 ± 0.48 ^hij^	19.09 ± 0.36 ^i^	27.38 ± 0.75 ^c^	1.96 ± 0.10 ^d^	5.69 ± 0.17
Zafiro	60.32 ± 2.08 ^abc^	33.93 ± 0.31 ^a^	24.25 ± 0.28 ^a^	37.15 ± 0.48 ^a^	3.34 ± 0.07 ^a^	5.52 ± 0.17
Environment (E)						
F2021	48.88 ± 1.41 ^c^	28.63 ± 0.34 ^b^	20.61 ± 0.26 ^bc^	24.72 ± 0.82 ^bc^	1.71 ± 0.09 ^bc^	6.13 ± 0.11
F2022	62.28 ± 0.54 ^b^	29.21 ± 0.55 ^b^	20.79 ± 0.26 ^b^	25.38 ± 0.71 ^b^	1.76 ± 0.08 ^b^	6.02 ± 0.14
F2023	53.77 ± 0.94 ^c^	30.27 ± 0.34 ^a^	21.55 ± 0.23 ^a^	24.20 ± 0.79 ^cd^	1.46 ± 0.08 ^d^	4.97 ± 0.08
NFI2021	62.67 ± 0.50 ^b^	26.35 ± 0.41 ^d^	19.25 ± 0.26 ^d^	23.11 ± 0.83 ^ef^	1.45 ± 0.09 ^d^	5.91 ± 0.13
NFI2022	62.87 ± 0.60 ^ab^	26.68 ± 0.40 ^d^	18.23 ± 0.23 ^e^	29.22 ± 0.72 ^a^	2.15 ± 0.07 ^a^	5.31 ± 0.14
NFI2023	63.84 ± 0.57 ^a^	27.67 ± 0.37 ^c^	20.38 ± 0.26 ^c^	23.70 ± 0.77 ^de^	1.55 ± 0.08 ^cd^	5.28 ± 0.10
*p*-value						
G	0.0299	0.0000	0.0000	0.0000	0.0000	0.5263
E	0.0000	0.0000	0.0000	0.0000	0.0000	0.9996
G x E	0.0298	0.0023	0.0000	0.0002	0.9980	1.0000

WG: whole-grain yield percentage; TGWPa: thousand-grain weight of paddy rice; TGWPo: thousand-grain weight of polished rice; CHA: grain chalkiness; TRAN: grain translucency; ADD: average degree of dispersion. Different letters in the same column indicate statistical differences according to the LSD Fisher test (*p* ≤ 0.05).

**Table 3:** Genotypic (above diagonal) and phenotypic (below diagonal) correlation among agronomic, productive, and quality traits of nineteen black rice genotypes and a white rice cultivar assessed under conventional flooding and NFI over three years (2021, 2022 and 2023).

Trait	GY	WG	DSA	PH	CHA	TRAN	PL	ST	FGP	TGP	TGWPa	TGWPo	ADD
GY		0.02	0.56	0.23	0.48	0.59	0.04	−0.84	0.46	−0.05	0.57	0.43	0.16
		ns	*	ns	*	**	ns	***	*	ns	**	ns	ns
WG	0.07		0.15	0.01	0.38	0.40	−0.20	−0.12	−0.63	−0.76	0.61	0.71	−0.09
	ns		ns	ns	ns	ns	ns	ns	**	***	**	***	ns
DSA	0.50	0.11		−0.02	−0.09	0.07	0.21	−0.53	0.05	−0.25	0.40	0.37	0.72
	*	ns		ns	ns	ns	ns	*	ns	ns	ns	ns	***
PH	0.32	−0.08	−0.05		0.43	0.45	0.20	−0.21	0.09	0.02	0.50	0.50	−0.06
	ns	ns	ns		ns	*	ns	ns	ns	ns	*	*	ns
CHA	0.43	0.32	−0.08	0.46		0.98	−0.14	−0.64	0.32	−0.04	0.74	0.74	−0.46
	ns	ns	ns	*		***	ns	**	ns	ns	***	***	*
TRAN	0.54	0.34	0.07	0.47	0.98		−0.09	−0.73	0.30	−0.12	0.82	0.80	−0.37
	*	ns	ns	*	***		ns	***	ns	ns	***	***	ns
PL	0.04	−0.12	0.16	0.23	−0.09	−0.05		0.40	−0.36	−0.21	0.10	0.14	0.53
	ns	ns	ns	ns	ns	ns		ns	ns	ns	ns	ns	*
ST	−0.65	0.06	−0.33	−0.12	−0.49	−0.54	0.16		−0.51	0.07	−0.67	−0.61	−0.13
	**	ns	ns	ns	*	ns	ns		*	ns	**	**	ns
FGP	0.43	−0.52	0.03	0.09	0.28	0.26	−0.15	−0.64		0.85	0.21	0.04	−0.18
	ns	*	ns	ns	ns	ns	ns	**		***	ns	ns	ns
TGP	−0.01	−0.60	−0.20	0.05	−0.03	−0.10	−0.09	0.01	0.75		−0.13	−0.30	−0.25
	ns	**	ns	ns	ns	ns	ns	ns	***		ns	ns	ns
TGWPa	0.54	0.43	0.36	0.44	0.75	0.82	0.12	−0.45	0.16	−0.12		1.00	−0.11
	*	ns	ns	ns	***	***	ns	*	ns	ns		***	ns
TGWPo	0.47	0.50	0.33	0.44	0.8	0.85	0.15	−0.40	0.04	−0.24	0.95		−0.09
	*	*	ns	ns	***	***	ns	ns	ns	ns	***		ns
ADD	0.15	−0.10	0.67	−0.10	−0.44	−0.36	0.39	−0.08	−0.13	−0.21	−0.14	−0.13	
	ns	ns	**	ns	ns	ns	ns	ns	ns	ns	ns	ns	

* *p* ≤ 0.05, ** *p* ≤ 0.01, and *** *p* ≤ 0.001. ns: not significant. GY: grain yield; WG: whole-grain yield percentage; DSA: days from sowing to anthesis; PH: plant height; CHA: grain chalkiness; TRAN: grain translucency; PL: panicle length; ST: sterility percentage; FGP: filled grain per panicle; TGP: total filled grain per panicle; TGWPa: thousand-grain weight of paddy rice; TGWPo: thousand-grain weight of polished rice; ADD: Average degree of dispersion. *n* = 60.

**Table 4:** Variance components and heritability of agronomic, productive, and quality traits of nineteen black rice genotypes and a white rice cultivar assessed under conventional flooding and non-flooding irrigation over three consecutive years.

	σ^2^_g_		σ^2^_env_		σ^2^_ge_		σ^2^_ε_	H^2^
GY	30.74	***	562.65	***	24.60	***	97.82	0.76
WG	7.22	***	35.71	***	17.80	***	14.30	0.66
DSA	13.38	***	183.51	***	1.39	ns	10.62	0.94
PH	39.62	***	76.18	***	12.99	***	28.10	0.91
CHA	29.48	***	3.36	ns	2.94	***	2.83	0.98
TRAN	0.36	***	0.06	**	0.03	***	0.04	0.98
PL	0.41	*	1.22	***	0.21	ns	6.32	0.51
ST	7.42	ns	171.65	***	19.06	**	100.99	0.46
FGP	13.4	**	120.67	***	10.4	ns	148.25	0.57
TGP	29.98	***	57.61	**	0.00	ns	213.23	0.72
TGWPa	4.70	***	2.11	***	0.99	**	4.52	0.92
TGWPo	2.95	***	1.38	***	0.31	***	0.66	0.97
ADD	0.48	***	0.22	***	0.05	*	0.36	0.94

* *p* ≤ 0.05, ** *p* ≤ 0.01, and *** *p* ≤ 0.001. ns: not significant. σ^2^_g_: genotype variance; σ^2^_env_: environment variance; σ^2^_ge_: genotype x environment variance; σ^2^_ε_: residual/error variance; H^2^: heritability. GY: grain yield; WG: whole grain yield percentage; DSA: days from sowing to anthesis; PH: plant height; CHA: grain chalkiness; TRAN: grain translucency; PL: panicle length; ST: sterility percentage; FGP: filled grain per panicle; TGP: total grain per panicle; TGWPa: thousand-grain weight of paddy rice; TGWPo: thousand-grain weight of polished rice; ADD: average degree of dispersion.

## Data Availability

All data were generated during the study. They are not publicly available but can be accessed through the following Supplementary Data.

## Supplementary Materials

The following supporting information can be downloaded at https://www.mdpi.com/article/10.3390/plants14223459/s1: Figure S1: Air temperature (maximum, minimum, and average) and accumulated rainfall recorded in San Carlos in (A)2020/21, (B) 2021/22, and (C) 2022/23 seasons. Figure S2: Linear correlation between days from sowing to anthesis (DSA) and accumulated degree days (DDA) of contrasting environments (traditional cultivation: flooding and non-flooding irrigation: NFI) for the (a) 2021, (b) 2022, and (c) 2023 seasons. Figure S3: Principal Component Analysis (PCA) biplot based on grain yield of nineteen black rice genotypes and one white rice cultivar evaluated under contrasting environments-conventional flooding (F) and non-flooding irrigation (NFI)-over three years (2021, 2022, and 2023). Figure S4: Principal Component Analysis (PCA) biplot of agronomic, productive, and quality traits of nineteen black rice genotypes evaluated in nineteen black rice genotypes and one white rice cultivar evaluated under contrasting environments-conventional flooding (F) and non-flooding irrigation (NFI)-over three years (2021, 2022, and 2023). Table S1: Analysis of variance of agronomic, productive, and quality traits evaluated in twenty rice genotypes under conventional flooding over three years (2021, 2022, and 2023). Table S2: Analysis of variance of agronomic, productive, and quality traits evaluated in twenty rice genotypes under non-flooding irrigation over three years (2021, 2022, and 2023). Table S3. Planting and harvesting dates for rice cultivated under flooded and non-flooded conditions in the 2021, 2022, and 2023 seasons. Table S4. Thermal amplitude and frequency of extreme temperature days during rice phenological stages. Table S5: Number of days below or above critical temperature thresholds during rice phenological stages under two irrigation regimes across three growing seasons (2021–2023).
