# Supplementary material for: Black Rice Performance Under Water Deficit Conditions and Genotype X Environment Interactions
Source: Plants (Basel). 2025 Nov 12;14(22):3459. doi: 10.3390/plants14223459 (PMC12655899; doi:10.3390/plants14223459)
Supplement: Supplementary file 1 [file plants-14-03459-s001.zip › plants-3922485-supplementary.pdf]

Supplementary Data

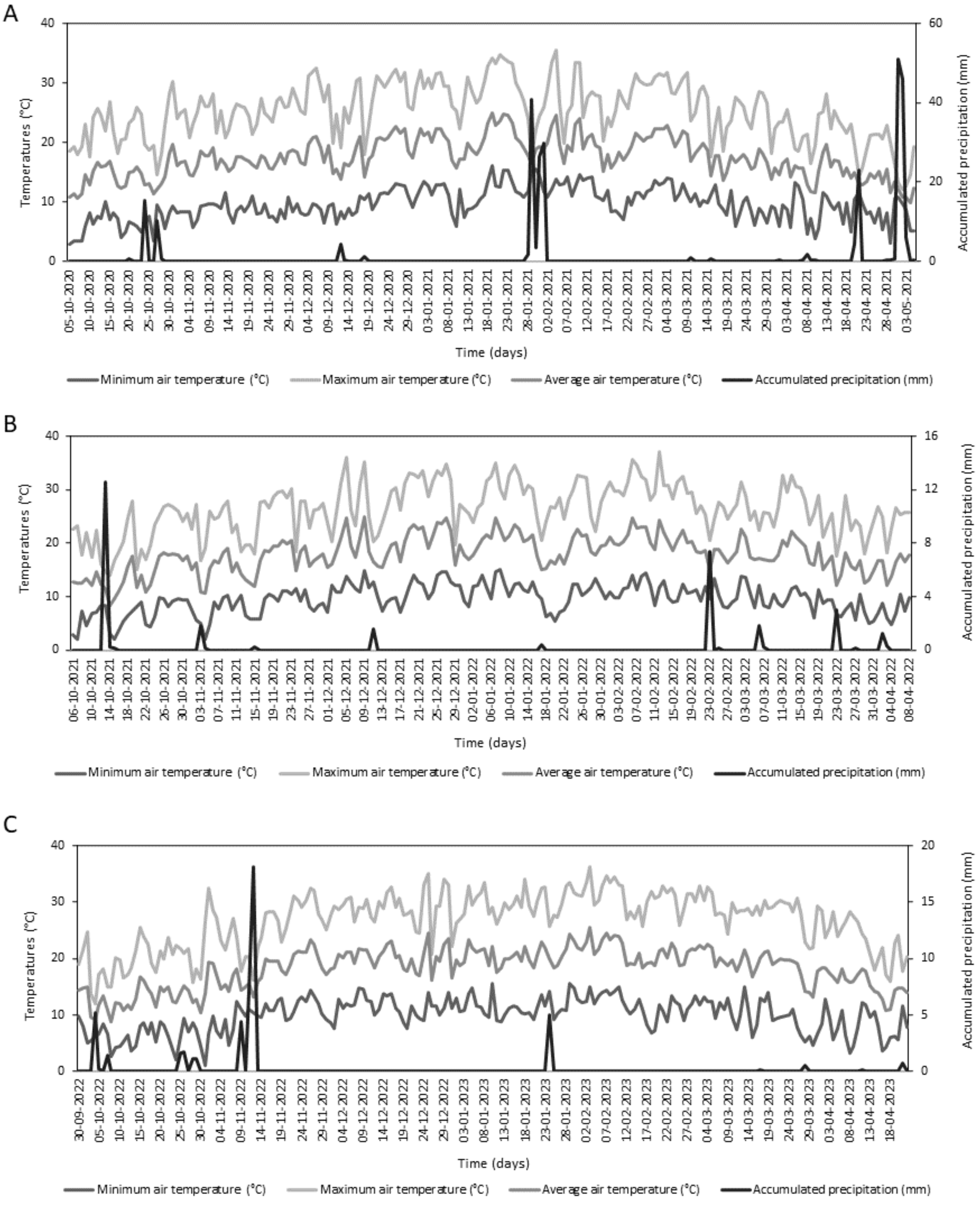

Figure S1. Air temperature (maximum, minimum, and average) and accumulated rainfall recorded in San Carlos in: A) 2020/21, B) 2021/22, and C) 2022/23 seasons.

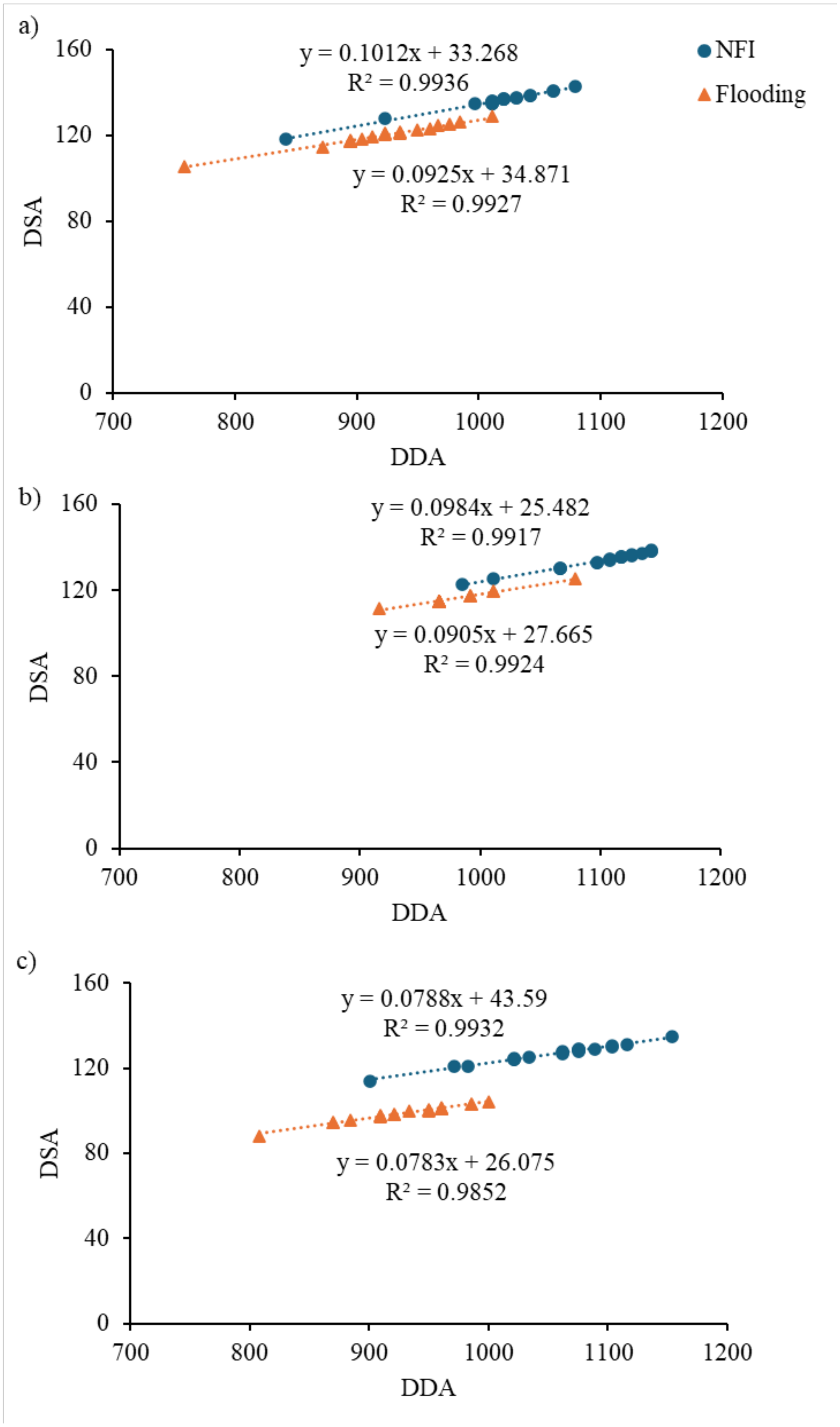

Figure S2. Linear correlation between days from sowing to anthesis (DSA) and accumulated degree days (DDA) of contrasting environments (traditional cultivation: flooding and non-flooding irrigation: NFI) for the a) 2021, b) 2022, and c) 2023 seasons.

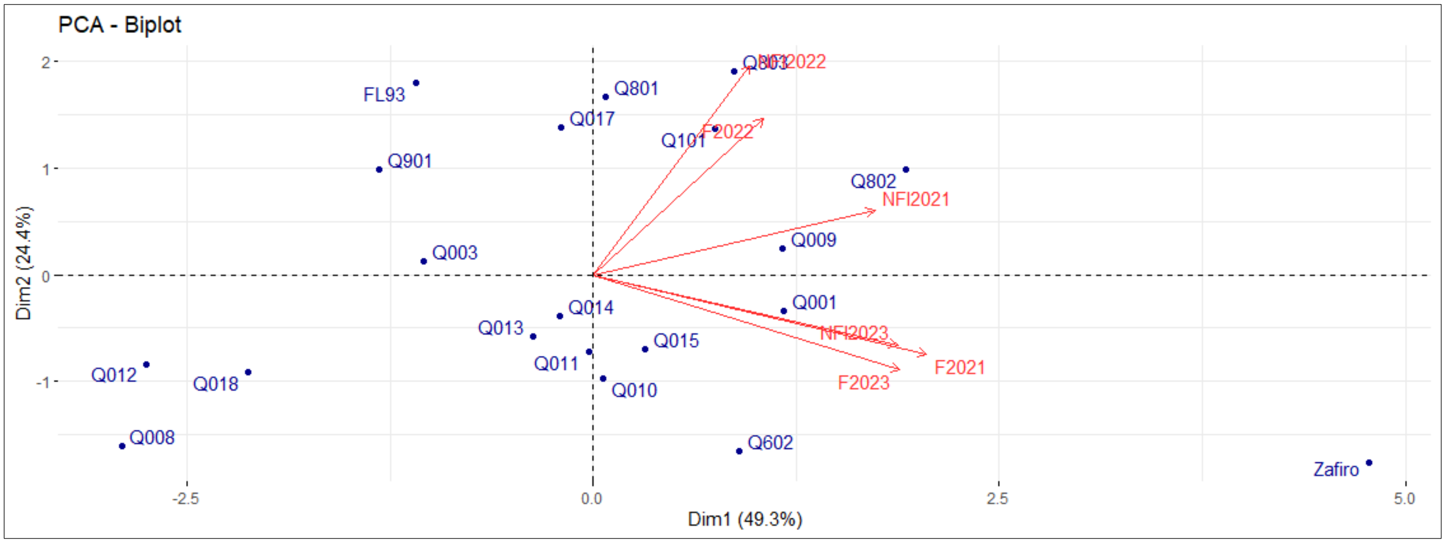

Figure S3. Principal Component Analysis (PCA) biplot based on grain yield of nineteen black rice genotypes and one white rice cultivar evaluated under contrasting environments - conventional flooding (F) and non-flooding irrigation (NFI) - over three years (2021, 2022, and 2023). Black rice genotypes: FQuila 93, FL93; Quila 291602, Q602; Quila 292001, Q001; Quila 292003, Q003; Quila 292008, Q008; Quila 292009, Q009; Quila 292010, Q010; Quila 292011, Q011; Quila 292012, Q012; Quila 292013, Q013; Quila 292014, Q014; Quila 292015, Q015; Quila 292017, Q017; Quila 292018, Q018; Quila 297901, Q901; Quila 279101, Q101; Quila 299801, Q801; Quila 299802, Q802; Quila 299803, Q803. White rice genotype: Zafiro.

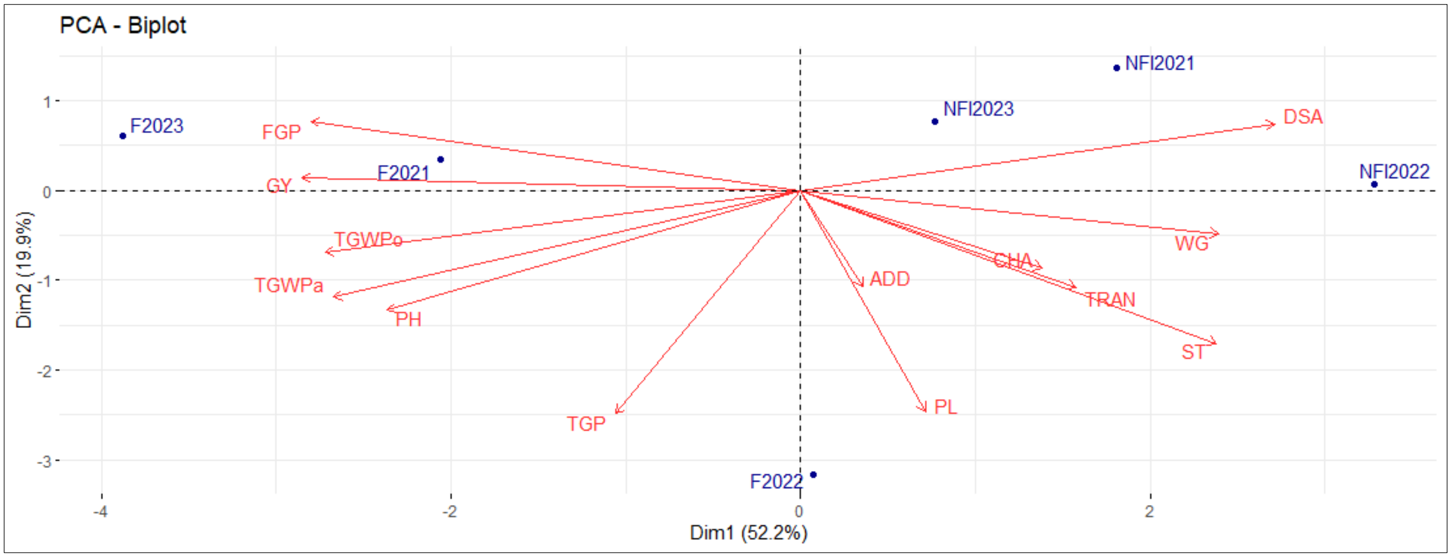

Figure S4. Principal Component Analysis (PCA) biplot of agronomic, productive, and quality traits of nineteen black rice genotypes evaluated in nineteen black rice genotypes and one white rice cultivar evaluated under contrasting environments - conventional flooding (F) and non-flooding irrigation (NFI) - over three years (2021, 2022, and 2023). GY: grain yield, PH: plant height, DSA: days from sowing to anthesis, WG: whole grain percentage, CHA: grain chalkiness, TRA: grain translucency, PL: panicle length, TGP: total grain per panicle, FGP: filled grain per panicle, ST: sterility percentage, TGWPa: thousand-grain weight of paddy rice, TGWPo: thousand-grain weight of polished rice, and ADD: average degree of dispersion.

Table S1. Analysis of variance of agronomic, productive, and quality traits evaluated in twenty rice genotypes under conventional flooding over three years (2021,2022, and 2023).

	DSA (days)	PH (cm)	GY (ton ha^{-1})	FGP	TGP	ST (%)	PL (cm)
**Genotypes (G)**							
FLQuila 93	112.22 ±2.78	98.94 ±2.94^{ab}	5.13 ±5.06^{cde}	53.56 ±3.23^{abcd}	83.51 ±5.80^{de}	33.54 ±4.90^{cde}	17.50 ±0.51^{efg}
Quila 279101	116.00 ±3.24	91.83 ±3.01^{cdef}	6.38 ±5.41^{b}	56.53 ±7.36^{abcd}	100.47 ±4.30^{a}	43.13 ±7.95^{ab}	19.34 ±0.44^{a}
Quila 291602	113.44 ±3.81	98.56 ±3.28^{b}	6.66 ±9.71^{b}	51.31 ±3.53^{abcde}	85.71 ±4.65^{bcde}	38.06 ±5.88a^{bcd}	16.27 ±0.62^{h}
Quila 292001	112.89 ±3.10	88.72 ±1.81^{defg}	6.80 ±7.02^{ab}	59.42 ±3.87^{ab}	88.96 ±4.13^{abcd}	31.66 ±5.79^{de}	17.66 ±0.37^{def}
Quila 292003	109.33 ±3.33	86.33 ±2.39^{fgh}	6.13 ±5.70^{bc}	61.38 ±4.19^{a}	97.13 ±6.24^{ab}	33.64 ±6.62^{cde}	17.51 ±0.56^{efg}
Quila 292008	113.11 ±3.11	80.00 ±1.30^{h}	4.46 ±4.91^{e}	49.82 ±3.52^{bcde}	77.93 ±2.79^{e}	34.84 ±5.56^{bcde}	16.96 ±0.58^{fgh}
Quila 292009	113.11 ±2.58	83.78 ±2.51^{gh}	6.65 ±8.32^{b}	45.53 ±3.17^{de}	78.24 ±1.37^{e}	41.49 ±4.38^{abc}	17.59 ±0.51^{def}
Quila 292010	111.89 ±2.93	87.94 ±1.30^{defg}	5.96 ±7.50^{bc}	60.20 ±7.01^{ab}	92.13 ±4.96^{abcd}	34.40 ±7.13^{bcde}	18.02 ±0.39^{bcde}
Quila 292011	112.44 ±2.88	98.83 ±2.86^{ab}	6.41 ±6.86^{b}	51.67 ±5.66^{abcde}	85.87 ±4.75^{bcde}	39.69 ±6.12^{abcd}	18.43 ±0.40^{bcd}
Quila 292012	112.67 ±3.20	87.33 ±2.13^{efg}	4.71 ±6.89^{de}	41.22 ±4.25^{e}	76.82 ±3.53^{e}	45.22 ±6.08^{e}	22.21 ±4.39^{cde}
Quila 292013	110.44 ±3.26	89.17 ±2.41^{defg}	5.89 ±7.76^{bcd}	57.38 ±4.26^{abc}	88.16 ±3.67^{abcd}	34.03 ±5.06^{bcde}	17.33 ±0.44^{efgh}
Quila 292014	113.78 ±3.52	92.72 ±2.48^{bcdef}	5.92 ±7.75^{bc}	49.87 ±5.66^{bcde}	84.29 ±4.81^{bcde}	39.83 ±7.30^{abcd}	17.99 ±0.39^{bcde}
Quila 292015	110.44 ±3.08	87.72 ±2.22^{defg}	6.29 ±7.97^{b}	50.73 ±4.39^{abcde}	85.80 ±3.53^{bcde}	40.14 ±4.94^{abcd}	18.04 ±0.37^{bcde}
Quila 292017	109.00 ±3.49	84.61 ±1.82^{gh}	5.90 ±4.30^{bcd}	58.31 ±8.14^{abc}	94.93 ±5.38^{abc}	39.10 ±8.43^{abcd}	16.60 ±0.57^{gh}
Quila 292018	109.89 ±2.95	88.06 ±2.05^{defg}	5.13 ±5.52^{cde}	58.38 ±5.21^{abc}	87.00 ±3.67^{bcde}	33.29 ±4.64^{cde}	17.73 ±0.46^{def}
Quila 297901	101.67 ±4.40	93.06 ±2.56^{bcde}	5.71 ±7.33^{bcd}	60.04 ±5.13^{ab}	92.04 ±4.74^{abcd}	35.22 ±4.61^{bcde}	17.66 ±0.54^{def}
Quila 299801	108.11 ±3.21	105.06 ±3.47^{a}	5.33 ±4.76^{cde}	47.82 ±4.14^{cde}	85.20 ±4.07^{bcde}	43.09 ±5.32^{ab}	19.01 ±1.20^{bcde}
Quila 299802	114.33 ±3.80	92.94 ±2.83^{bcde}	6.73 ±7.31^{ab}	54.89 ±7.68^{abcd}	88.93 ±5.06^{abcd}	39.17 ±8.17^{abcd}	19.40 ±0.40^{a}
Quila 299803	110.11 ±3.04	94.06 ±2.07^{bcd}	5.96 ±4.87^{bcd}	61.51 ±6.44^{a}	94.44 ±6.66^{abcd}	34.92 ±4.60^{bcde}	18.56 ±0.31^{abc}
Zafiro	119.33 ±3.72	96.22 ±3.47^{bc}	8.54 ±12.22^{a}	60.27 ±5.49^{ab}	81.78 ±2.03^{cde}	26.41 ±6.37^{e}	18.73 ±0.37^{ab}
**Years (Y)**							
2021	120.13 ±0.77^{a}	95.25 ±1.50^{a}	7.12 ±2.38^{b}	54.49 ±1.40^{b}	80.63 ±1.19^{c}	32.46 ±1.40^{b}	16.92 ±0.19^{c}
2022	116.15 ±0.60^{a}	90.78 ±0.96^{b}	3.62 ±1.57^{a}	41.26 ±2.02^{c}	96.03 ±2.26^{a}	57.73 ±1.62^{a}	19.97 ±0.67^{a}
2023	98.85 ±0.50^{b}	87.86 ±1.01^{c}	7.37 ±1.93^{b}	67.72 ±1.58^{a}	85.74 ±1.57^{b}	20.94 ±1.21^{c}	17.48 ±0.24^{b}
**p-value**							
G	0.0927	0.0000	0.0000	0.0069	0.0046	0.0189	0.0000
Y	0.0000	0.0000	0.0000	0.0000	0.0000	0.0000	0.0000
G x Y	0.6977	0.0210	0.1064	0.0463	0.6339	0.0270	0.0002

	WG (%)	CHA	TRAN	TGWPa (g)	TGWPo (g)	ADD
**Genotypes (G)**						
FLQuila 93	61.97 ±0.62[a]	22.71 ±1.04[hi]	1.48 ±0.12[ij]	31.74 ±0.45[b]	22.91 ±0.47[b]	5.84 ±0.25[cde]
Quila 279101	44.64 ±4.81[fg]	12.81 ±0.55[m]	0.52 ±0.03[n]	28.69 ±0.67[cde]	18.61 ±0.31[i]	5.96 ±0.25[bcd]
Quila 291602	60.24 ±2.64[a]	34.01 ±0.72[b]	2.68 ±0.08[b]	32.28 ±0.75[ab]	24.12 ±0.18[a]	4.89 ±0.18[f]
Quila 292001	53.02 ±3.41[de]	27.37 ±0.51[c]	1.94 ±0.06[d]	29.02 ±0.68[cd]	21.37 ±0.22[cd]	6.19 ±0.24[ab]
Quila 292003	52.44 ±3.51[def]	26.62 ±0.67[cd]	1.84 ±0.10[def]	30.12 ±0.87[bc]	21.61 ±0.31[c]	6.06 ±0.22[abcd]
Quila 292008	54.09 ±3.01[de]	17.92 ±0.43[kl]	0.93 ±0.03[m]	27.07 ±0.80[def]	19.12 ±0.40[hi]	5.96 ±0.24[abcd]
Quila 292009	61.84 ±1.60[a]	17.64 ±0.77[l]	1.04 ±0.10[lm]	26.24 ±0.66[ef]	18.64 ±0.18[hi]	6.12 ±0.22[abc]
Quila 292010	52.56 ±4.11[de]	27.32 ±0.72[c]	1.78 ±0.06[defg]	32.14 ±2.42[b]	20.53 ±0.36[ef]	6.16 ±0.17[abc]
Quila 292011	59.97 ±1.71[ab]	24.69 ±0.53[efg]	1.71 ±0.06[efgh]	31.62 ±0.65[b]	22.64 ±0.26[b]	6.07 ±0.22[abcd]
Quila 292012	59.99 ±1.25[abc]	23.79 ±0.97[fgh]	1.58 ±0.10[hij]	30.57 ±0.73[bc]	22.98 ±0.34[b]	5.99 ±0.24[abcd]
Quila 292013	53.36 ±2.59[def]	19.61 ±0.37[jk]	1.06 ±0.03[klm]	27.45 ±0.38[def]	19.26 ±0.45[hi]	5.71 ±0.24[de]
Quila 292014	56.41 ±2.17[bcd]	25.26 ±0.78[def]	1.66 ±0.10[fghi]	28.41 ±0.70[cdef]	20.87 ±0.33[cde]	6.10 ±0.26[abc]
Quila 292015	51.85 ±2.99[def]	25.03 ±0.58[def]	1.61 ±0.08[ghi]	28.27 ±0.43[cdef]	20.72 ±0.32[def]	5.79 ±0.28[cde]
Quila 292017	50.46 ±3.51[def]	22.83 ±1.07[hi]	1.22 ±0.05[k]	27.10 ±1.07[def]	19.40 ±0.38[gh]	5.23 ±0.34[ef]
Quila 292018	51.76 ±2.33[ef]	21.29 ±0.70[ij]	1.11 ±0.07[kl]	26.65 ±0.41[ef]	18.83 ±0.25[hi]	5.67 ±0.27[de]
Quila 297901	56.06 ±2.11[cde]	33.42 ±0.72[b]	2.34 ±0.13[c]	30.33 ±0.34[bc]	21.23 ±0.18[cde]	2.92 ±0.23[g]
Quila 299801	53.55 ±1.62[def]	26.23 ±0.77[cde]	1.82 ±0.13[def]	28.59 ±0.62[cde]	20.94 ±0.20[cde]	5.70 ±0.29[cde]
Quila 299802	60.91 ±2.38[a]	23.16 ±1.14[gh]	1.40 ±0.12[j]	29.07 ±0.68[cd]	20.92 ±0.26[cde]	6.30 ±0.23[a]
Quila 299803	44.58 ±2.04[g]	26.44 ±0.82[cd]	1.88 ±0.11[de]	27.49 ±0.50[def]	20.07 ±0.51[fg]	5.63 ±0.25[de]
Zafiro	59.84 ±3.12[a]	37.13 ±0.79[a]	3.33 ±0.13[a]	34.56 ±0.39[a]	24.83 ±0.26[a]	5.86 ±0.23[cde]
**Years (Y)**						
2021	48.88 ±1.41[c]	24.72 ±0.82[ab]	1.71 ±0.09[a]	28.63 ±0.34[b]	20.61 ±0.26[b]	6.13 ±0.11[a]
2022	62.28 ±0.54[a]	25.38 ±0.71[a]	1.76 ±0.08[a]	29.21 ±0.55[b]	20.79 ±0.26[b]	6.02 ±0.14[a]
2023	53.77 ±0.94[b]	24.20 ±0.79[b]	1.46 ±0.08[b]	30.27 ±0.34[a]	21.55 ±0.23[a]	4.97 ±0.08[b]
**p-value**						
G	0.0000	0.0000	0.0000	0.0000	0.0000	0.0000
Y	0.0000	0.0032	0.0000	0.0016	0.0000	0.0000
G x Y	0.0000	0.0000	0.0000	0.1281	0.0002	0.0000

GY: grain yield, PH: plant height, DSA: days from sowing to anthesis, WG: whole grain percentage, CHA: grain chalkiness, TRA: grain translucency, PL: panicle length, TGP: total grain per panicle, FGP: filled grain per panicle, ST: sterility percentage, TGWPa: thousand-grain weight of paddy rice, TGWPo: thousand-grain weight of polished rice, and ADD: average degree of dispersion. Different letters in the same column indicate statistical differences according to the LSD Fischer test ($p \leq 0.05$). n = 180.

Table S2. Analysis of variance of agronomic, productive, and quality traits evaluated in twenty rice genotypes under non-flooding irrigation over three years (2021, 2022, and 2023).

	DSA (days)	PH (cm)	GY (ton ha^{-1})	FGP	TGP	ST (%)	PL (cm)
**Genotypes (G)**							
FLQuila 93	134.89 ±1.95	84.50 ±1.41	2.50 ±1.83	43.96 ±2.15	76.09 ±3.03^{abcdef}	41.79 ±2.76^{cde}	17.65 ±0.31^{cd}
Quila 279101	134.67 ±1.72	78.50 ±1.50	2.75 ±1.70	42.47 ±4.90	82.82 ±6.43^{abc}	48.51 ±4.81^{abcd}	18.61 ±0.30^{b}
Quila 291602	133.67 ±1.37	81.83 ±2.18	2.53 ±4.24	43.89 ±5.05	78.53 ±8.67^{abcde}	43.36 ±2.58^{bcde}	16.28 ±0.40^{f}
Quila 292001	132.56 ±1.93	72.50 ±1.93	2.65 ±3.63	40.53 ±5.34	66.31 ±6.79^{def}	39.22 ±4.03^{de}	17.47 ±0.42^{cde}
Quila 292003	132.33 ±2.32	66.50 ±3.75	1.89 ±2.66	41.44 ±3.86	76.02 ±6.44^{abcdef}	44.23 ±3.40^{bcd}	19.12 ±1.85^{cde}
Quila 292008	133.89 ±1.64	68.78 ±2.79	1.83 ±4.13	40.02 ±2.02	79.51 ±2.93^{abcde}	49.52 ±2.16^{abc}	17.38 ±0.27^{cde}
Quila 292009	133.44 ±1.33	63.50 ±1.00	2.83 ±2.42	32.24 ±4.56	60.80 ±7.70^{f}	47.70 ±3.48^{abcd}	16.91 ±0.22^{ef}
Quila 292010	131.78 ±1.95	75.94 ±2.85	2.58 ±3.56	39.20 ±2.84	83.38 ±4.01^{abc}	51.16 ±5.20^{abc}	17.61 ±0.28^{cde}
Quila 292011	136.22 ±1.62	80.94 ±0.90	2.18 ±2.48	39.41 ±2.94	79.44 ±3.52^{abcde}	50.21 ±3.34^{abc}	18.67 ±0.30^{b}
Quila 292012	130.89 ±1.22	72.83 ±1.80	1.79 ±1.68	29.49 ±3.68	61.04 ±4.22^{f}	51.08 ±5.06^{abc}	17.57 ±0.22^{cde}
Quila 292013	131.78 ±1.68	70.44 ±1.54	2.39 ±3.23	44.62 ±5.12	78.89 ±5.33^{abcde}	44.47 ±3.04^{bcd}	17.36 ±0.23^{def}
Quila 292014	133.11 ±1.57	76.17 ±2.21	2.49 ±1.92	42.60 ±2.17	87.58 ±3.63^{ab}	50.47 ±3.29^{abc}	18.03 ±0.40^{bc}
Quila 292015	133.67 ±1.57	75.89 ±1.67	2.53 ±1.87	37.64 ±5.29	65.31 ±7.43^{ef}	43.28 ±3.82^{bcde}	17.72 ±0.23^{cd}
Quila 292017	128.89 ±2.06	68.33 ±2.27	2.47 ±2.88	53.80 ±6.25	92.06 ±7.19^{a}	42.32 ±2.79^{bcde}	17.77 ±0.53^{bcd}
Quila 292018	131.44 ±2.07	74.06 ±2.62	1.94 ±3.39	37.84 ±3.27	78.98 ±4.43^{abcde}	51.48 ±3.76^{ab}	18.40 ±0.23^{b}
Quila 297901	118.33 ±1.27	74.72 ±0.58	1.92 ±1.29	37.00 ±2.31	82.20 ±2.79^{abcd}	54.93 ±2.54^{a}	17.43 ±0.46^{cde}
Quila 299801	124.56 ±1.27	96.17 ±2.28	3.01 ±1.96	42.25 ±4.49	69.27 ±5.39^{cdef}	39.12 ±3.98^{de}	17.66 ±0.45^{cd}
Quila 299802	132.56 ±1.50	75.78 ±1.13	3.16 ±2.72	45.42 ±2.30	75.49 ±3.26^{bcdef}	39.85 ±1.76^{de}	19.91 ±0.32^{a}
Quila 299803	133.44 ±1.36	82.11 ±2.09	2.97 ±3.42	42.82 ±5.52	79.91 ±5.59^{abcde}	46.88 ±4.92^{abcd}	19.66 ±0.26^{a}
Zafiro	138.89 ±1.16	81.33 ±1.94	3.54 ±5.35	48.67 ±3.14	73.73 ±2.17^{bcdef}	34.15 ±3.41^{e}	18.05 ±0.25^{bc}
**Years (Y)**							
2021	135.75 ±0.71	74.54 ±1.46	2.03 ±1.24	42.08 ±1.22	78.93 ±1.24	46.28 ±1.53^{b}	17.06 ±0.15^{c}
2022	133.72 ±0.64	73.69 ±0.89	2.21 ±0.75	38.39 ±1.23	77.77 ±1.76	50.45 ±1.31^{a}	18.12 ±0.15^{b}
2023	126.68 ±0.67	79.89 ±1.08	3.25 ±1.26	43.32 ±2.36	72.41 ±3.35	40.33 ±1.55^{c}	18.71 ±0.31^{a}
**p-value**							
G	0.6254	0.9321	0.9893	0.0848	**0.0109**	**0.0014**	**0.0000**
Y	0.8930	0.9944	0.6043	0.0952	0.0959	**0.0000**	**0.0000**
G x Y	1.0000	1.0000	1.0000	0.4525	0.7957	**0.0461**	**0.2693**

	WG (%)	CHA	TRAN	TGWPa (g)	TGWPo (g)	ADD
**Genotypes (G)**						
FLQuila 93	64.76 ±0.53^{abc}	23.07 ±1.52^{fg}	1.62 ±0.15^{gh}	29.14 ±0.68^{bc}	21.60 ±0.28^{b}	5.92 ±0.15^{abcd}
Quila 279101	60.41 ±1.31^{ef}	13.78 ±1.68^{i}	0.62 ±0.11^{l}	25.57 ±0.83^{efg}	17.80 ±0.65^{ghi}	6.12 ±0.25^{a}
Quila 291602	66.13 ±0.63^{a}	32.86 ±0.44^{b}	2.49 ±0.06^{b}	30.68 ±0.34^{b}	21.63 ±0.41^{b}	4.32 ±0.35^{g}
Quila 292001	63.56 ±1.65^{abc}	26.63 ±1.30^{d}	1.73 ±0.16^{fgh}	29.03 ±0.98^{bc}	19.32 ±0.32^{de}	5.83 ±0.13^{abcde}
Quila 292003	65.76 ±1.10^{a}	27.34 ±1.31^{d}	1.90 ±0.13^{def}	27.19 ±0.87^{de}	19.42 ±0.28^{d}	5.79 ±0.20^{abcde}
Quila 292008	63.71 ±0.72^{bcde}	18.26 ±1.41^{h}	0.99 ±0.12^{k}	24.51 ±0.50^{gh}	17.51 ±0.61^{hi}	5.46 ±0.19^{def}
Quila 292009	65.60 ±0.85^{ab}	19.13 ±1.33^{h}	1.23 ±0.18^{jk}	24.26 ±0.55^{gh}	17.17 ±0.46^{i}	5.82 ±0.24^{abcde}
Quila 292010	65.32 ±0.87^{ab}	27.27 ±1.13^{d}	1.89 ±0.14^{def}	26.37 ±1.05^{ef}	19.34 ±0.57^{de}	5.67 ±0.19^{bcdef}
Quila 292011	61.78 ±1.62^{cdef}	28.09 ±1.16^{cd}	2.07 ±0.15^{cd}	29.46 ±0.64^{bc}	21.32 ±0.47^{b}	5.67 ±0.27^{abcde}
Quila 292012	65.28 ±1.08^{ab}	23.63 ±1.66^{ef}	1.51 ±0.17^{hi}	27.21 ±0.42^{de}	20.36 ±0.39^{c}	5.53 ±0.21^{cdef}
Quila 292013	62.44 ±0.91^{cdef}	21.06 ±1.61^{fgh}	1.20 ±0.14^{jk}	25.05 ±0.40^{fgh}	18.00 ±0.51^{fghi}	5.37 ±0.26^{ef}
Quila 292014	64.17 ±1.01^{abc}	26.54 ±1.28^{d}	1.80 ±0.11^{efg}	25.51 ±0.33^{efg}	18.77 ±0.27^{def}	6.09 ±0.21^{ab}
Quila 292015	63.08 ±1.22^{bcde}	26.26 ±0.88^{de}	1.73 ±0.09^{fgh}	25.90 ±1.12^{efg}	19.24 ±0.48^{de}	5.84 ±0.17^{abcde}
Quila 292017	58.47 ±1.81^{f}	22.66 ±1.62^{fg}	1.30 ±0.16^{ij}	23.58 ±0.59^{h}	17.52 ±0.24^{hi}	5.21 ±0.33^{ef}
Quila 292018	62.20 ±1.24^{cdef}	20.48 ±1.91^{gh}	1.15 ±0.18^{jk}	25.13 ±0.60^{fgh}	17.50 ±0.49^{hi}	5.70 ±0.23^{abcde}
Quila 297901	63.88 ±0.71^{bcd}	30.90 ±1.02^{bc}	2.18 ±0.14^{c}	26.37 ±0.45^{ef}	18.58 ±0.23^{efg}	2.94 ±0.46^{g}
Quila 299801	61.20 ±1.07^{def}	26.62 ±1.65^{d}	1.69 ±0.18^{fgh}	25.95 ±0.82^{efg}	18.39 ±0.31^{fg}	5.77 ±0.15^{abcde}
Quila 299802	63.83 ±0.94^{bcd}	26.78 ±1.43^{d}	1.81 ±0.16^{efg}	28.75 ±0.30^{cd}	20.42 ±0.53^{c}	5.98 ±0.23^{abc}
Quila 299803	60.16 ±1.39^{ef}	28.32 ±1.17^{cd}	2.05 ±0.15^{cde}	25.03 ±0.58^{fgh}	18.11 ±0.22^{fgh}	5.76 ±0.21^{abcde}
Zafiro	60.79 ±2.74^{bcd}	37.17 ±0.56^{a}	3.36 ±0.04^{a}	33.30 ±0.37^{a}	23.67 ±0.41^{a}	5.19 ±0.18^{fg}
**Years (Y)**						
2021	62.67 ±0.50^{b}	23.11 ±0.83^{b}	1.45 ±0.09^{c}	26.35 ±0.41^{b}	19.25 ±0.26^{b}	5.91 ±0.13^{a}
2022	62.87 ±0.60^{b}	23.70 ±0.72^{a}	2.15 ±0.07^{a}	26.68 ±0.40^{b}	18.23 ±0.23^{c}	5.31 ±0.14^{b}
2023	63.84 ±0.57^{a}	29.22 ±0.77^{b}	1.55 ±0.08^{b}	27.67 ±0.37^{a}	20.38 ±0.26^{a}	5.28 ±0.10^{b}
**p-value**						
G	**0.0000**	**0.0000**	**0.0000**	**0.0000**	**0.0000**	**0.0000**
Y	**0.0108**	**0.0000**	**0.0000**	**0.0003**	**0.0000**	**0.0000**
G x Y	**0.0000**	0.2165	**0.0083**	**0.0009**	**0.0031**	**0.0062**

GY: grain yield, PH: plant height, DSA: days from sowing to anthesis, WG: whole grain percentage, CHA: grain chalkiness, TRA: grain translucency, PL: panicle length, TGP: total grain per panicle, FGP: filled grain per panicle, ST: sterility percentage, TGWPa: thousand-grain weight of paddy rice, TGWPo: thousand-grain weight of polished rice, and ADD: average degree of dispersion. Different letters in the same column indicate statistical differences according to the LSD Fischer test ($p \leq 0.05$). n = 180.

Table S3. Planting and harvesting dates for rice cultivation under flooded and non-flooded irrigation regimes during the 2021, 2022, and 2023 growing seasons.

Season	Water regime	Planting	Harvesting
2021	Flooding (F)	05-10-2020	05-05-2021
	Non flooding irrigation (NFI)	25-09-2020	06-04-2021
2022	Flooding (F)	12-10-2021	29-03-2022
	Non flooding irrigation (NFI)	06-10-2021	08-04-2022
2023	Flooding (F)	25-10-2022	22-04-2023
	Non flooding irrigation (NFI)	30-09-2022	22-04-2023

Table S4. Thermal amplitude and frequency of extreme temperature days during rice phenological stages.

Season	Water management	Phenological stage	Duration (days)	Days with ΔT < 7–8 °C	Days with ΔT > 18 °C	Occurrences of ≥3 consecutive days with ΔT > 18 °C
2020-2021	Flooding	Sowing	64	1	19	2
		Active tillering	15	0	10	2
		Panicle initiation	31	0	20	4
		Flowering to harvest	103	12	26	3
2020-2021	Non-flooding irrigation	Sowing	76	2	21	2
		Active tillering	23	0	14	3
		Panicle initiation	31	2	17	3
		Flowering to harvest	64	4	21	2
2021-2022	Flooding	Sowing	64	3	18	1
		Active tillering	15	0	13	1
		Panicle initiation	31	0	14	4
		Flowering to harvest	59	0	26	4
2021-2022	Non-flooding irrigation	Sowing	76	3	26	2
		Active tillering	23	0	13	3
		Panicle initiation	31	0	18	4
		Flowering to harvest	55	0	21	2
2022-2023	Flooding	Sowing	64	2	23	3
		Active tillering	15	0	8	2
		Panicle initiation	31	0	24	3
		Flowering to harvest	70	2	37	6
2022-2023	Non-flooding irrigation	Sowing	76	3	18	2
		Active tillering	23	0	12	2
		Panicle initiation	31	0	22	3
		Flowering to harvest	75	2	42	7

ΔT represents the daily thermal amplitude (°C), calculated as the difference between the maximum and minimum temperatures. Values were obtained from the DELTA index analysis conducted throughout the rice growing season.

Table S5. Number of days below or above critical temperature thresholds during rice phenological stages under flooded and NFI irrigation regimes across three growing seasons (2021–2023).

Phenological stage	Water management	Critical temperatures	2020-2021	2021-2022	2022-2023
Sowing	Flooding	Tmin < 12 °C	64	59	46
		Tmax > 30°C	4	5	14
		Tmax > 37 °C	0	0	0
	NFI	Tmin < 12 °C	76	70	61
		Tmax > 30°C	4	9	9
		Tmax > 37 °C	0	0	0
Active tillering	Flooding	Tmin < 12 °C	15	7	10
		Tmax > 30°C	4	11	6
		Tmax > 33 °C	0	4	3
	NFI	Tmin < 12 °C	18	8	16
		Tmax > 30°C	10	14	9
		Tmax > 33 °C	0	33	3
Panicle initiation	Flooding	Tmin < 18 °C	31	31	31
		Tmax > 30°C	16	12	19
		Tmax > 35 °C	0	0	1
	NFI	Tmin < 18 °C	31	31	31
		Tmax > 30°C	12	12	16
		Tmax > 35 °C	0	0	1
Flowering to harvest	Flooding	Tmin < 22 °C	103	59	70
		Tmax > 30°C	14	17	16
		Tmax > 33 °C	6	3	0
	NFI	Tmin < 22 °C	64	55	75
		Tmax > 30°C	12	9	21
		Tmax > 33 °C	4	0	4

Tmin and Tmax represent the daily minimum and maximum air temperatures, respectively. Critical thresholds indicate stress conditions during sensitive stages of rice development, based on temperature limits established in the literature.
